# All-atom molecular dynamics simulation of Cyclophilin A in complex with Sanglifehrin A

**DOI:** 10.1371/journal.pone.0358847

**Published:** 2026-09-22

**Authors:** Zahra Aliabadi, Alireza Mohebbi

**Affiliations:** 1 Student Research Committee, School of Medicine, Golestan University of Medical Sciences, Gorgan, Iran; 2 Department of Virology, Iran University of Medical Sciences, Tehran, Iran; 3 Vista Aria Rena Gene Inc., Gorgan, Iran; University of Rajshahi, BANGLADESH

## Abstract

Cyclophilin A (CypA), a key peptidyl-prolyl cis/trans isomerase, plays a pivotal role in various biological processes and is a target for therapeutic intervention, particularly in viral and cancer-related diseases. This study explores the structural and dynamic effects of Sanglifehrin A (SangfA), a potent CypA inhibitor, using molecular dynamics (MD) simulation. All-atom MD simulations (100 ns) of apo CypA and its complex with SangfA were performed using GROMACS v2023. In addition to MD analyses, essential dynamics, clustering, secondary structure, and binding free energy were analyzed. SangfA binding induces a dual dynamic response: local rigidification of the active-site β2-β3 loop, residues 100–102 and 108–111 with a coil‑to‑β-strand transition (β-sheet content rises from 28.0% to 32.3%), alongside enhanced mobility in distal loops and a 1.1‑fold expansion of the conformational ensemble. Principal component analysis shows redistribution of motions from a single dominant mode (apo PC1 = 44.2%) to more diffuse fluctuations (holo PC1 = 30.4%), increasing configurational entropy. The ligand forms a dynamic hydrogen‑bond network (1–2 persistent bonds) anchored by Arg55 and hydrophobic contacts with Phe60 and Lys105, while Arg55 remains uninvolved. The computed binding free energy (ΔG = −20.45 ± 3.07 Kcal/mol) accords with picomolar potency. SangfA acts via conformational selection, stabilizing a pre‑existing CypA substate with augmented β-structure and broader dynamics. This entropically driven mechanism challenges static inhibition paradigms. Future efforts should validate the β2-β3 loop transition experimentally and exploit the Lys105 contact to design next‑generation cyclophilin inhibitors.

## Introduction

Cyclophilins (Cyps) are peptidyl-prolyl cis/trans isomerases (PPIase) in prokaryotic and eukaryotic cells. Cyps catalyzes the interconversion of the *cis* and *trans* isomers of the planar peptide bond preceding an internal proline residue. Cyps are also known as immunophilins owing to their interactions with cyclosporine A (CsA), a cyclic peptide derived from the fungus *Tolypocladium inflatum* [1]. Cyps share a common domain of 109 amino acids, the cyclophilin-like domain, surrounded by domains unique to each family member and associated with their subcellular compartmentalization and functional specialization. They also adopt an eight-stranded anti-parallel P-barrel structure. Seventeen human Cyps have been identified thus far. Cyclophilin A (CypA) is the most abundant Cyp presented in the cytosol and is involved in protein folding and trafficking, immunomodulation, cell signaling, and cell death. Further cellular pathways regulated by CypA are reviewed by Dawar et al. [2].

CypA is involved in several human-associated complications, including Rheumatoid arthritis (RA) [3], Cardiovascular disease, Hepatocellular carcinoma (HCC) [4, 5], and pancreatic and lung cancers [3]. Moreover, CypA regulates the pathogenesis of several DNA or RNA viruses, including Human Immunodeficiency virus (HIV) [4,5], Coronaviruses (CoVs) like SARS-CoV-2 [6], Flaviviruses [7] like Dengue virus (DENV), Japanese Encephalitis virus (JEV), Yellow Fever virus (YFV), and Hepatitis C virus (HCV) [8], Hepatitis B virus (HBV) [9,10], human Cytomegalovirus (hCMV) [11], Influenza A virus (IAV) [12], Enteroviruses [13], Vaccinia virus (VV) [14], and Vesicular Stomatitis virus (VSV) [15]. It has been reported that CypA escalated an opportunistic viral and bacterial co-infection [16]. Furthermore, Li et al. reported the role of CypA in the inflammatory pathogenesis of *Mycoplasma genitalium* through activation of signal-regulated kinases (ERK) phosphorylation and NF-kB [17]. The findings indicate the potential of CypA as a target for developing viral and bacterial agents. Besides CsA and CsA-derivatives, some structurally divergent inhibitors with improved anti-viral effects present higher affinity to CypA. Sanglifehrins, including SangfA, SFB, and SFC, are interesting in multidrug-resistant viral infections [18]. CsA and non-immunosuppressive macrocyclic analogs of CsA and SangfA potently inhibit cyclophilin PPIase activity by binding its catalytic site. Developing CypA inhibitors aimed at anti-viral or cancer therapeutics is undertaken to obtain chemicals with improved drug-likeness, low toxicity, and higher efficiency. Li et al. employed the structure-activity relationship (SAR) and molecular docking of seven bisamide derivatives. They showed that derivative 7c is an effective CypA inhibitor for anti-HCV treatment [18]. Thus, CypA represents an attractive target for the development of novel inhibitors.

Some inhibitors show adverse effects due to their off-target activities. In this regard, it has been demonstrated that CsA and its derivatives suppress the mitogen-activated protein kinase pathway, affect transforming growth factor-b1 levels and ABC transporters, and enhance major histocompatibility complex-I surface expression [19–23]. Furthermore, the phase III clinical trial of Alisporivir has stopped due to fatal acute pancreatitis cases in HCV cases with an unrecognized mechanism [24]. Ahmed-Belkacem et al. utilized molecular docking, NMR, and X-ray crystallography for fragment-based drug discovery (FBDD) of novel non-peptidic, small-molecule cyclophilin inhibitors unrelated to immunosuppressor CypA inhibitors with potent PPIase inhibitory activity. The fragment growing strategy has led to the discovery of some potent CypA inhibitors with antiviral effectiveness against HCV, HIV, and coronaviruses in vitro [25].

Unlike rigid protein conformations in traditional molecular docking, MD simulation provides flexible protein conformations in complex with ligands during a certain simulation time [26]. MD simulation has evolved from a specialized computational technique into a cornerstone of modern biomedical research and drug discovery [27,28]. The historical foundation of biological MD was established nearly five decades ago from static structures to currently dynamic entities [29,30]. Since then, the field has diversified into multiple methodological categories, including all-atom MD, where atoms are treated as charged spheres governed by classical mechanics. The reliability of these simulations critically depends on force field selection, with rigorously tested parameter sets implemented in widely adopted software packages such as GROMACS, AMBER, and DESMOND demonstrating consistent performance across diverse biological applications [27]. These techniques are now being utilized for evaluating the dynamic changes of a therapeutic receptor targeted by a ligand and drug discovery.

Accordingly, despite the therapeutic relevance of CypA inhibitors, the precise molecular mechanism by which SangfA achieves its potency and influences CypA dynamics remains incompletely understood. In particular, the extent to which ligand binding alters secondary structure elements, loop flexibility, and the global conformational landscape of CypA has not been characterized at an atomic level. In this study, we address this gap by performing an all-atom MD simulations of both apo CypA and its complex with SangfA. By conducting the study, the findings will provide a dynamic framework for the rational design of next-generation CypA inhibitors.

## Materials and methods

### Preparation of CypA crystal structure and its complex with SangfA

The crystallographic structure of human CypA (PDB ID: 1BCK) in complexed with 11 Cyclosporin derivatives and with the resolution of 1.8Å was provided by The Protein Data Bank (http://www.rcsb.org/pdb) [31,32]. CypA structure, as the receptor, cleaned and certain chains containing the molecule kept for continue using UCSF-Chimera 1.10.2 [33]. 3D chemical structure of known CypA inhibitor SangfA was obtained from a small molecule database, PubChem [9,34]. All structure’s energy was minimized using Swiss-pdb Viewer [35]. The complex of CypA-SangfA was constructed as described before [36,37].

### Molecular dynamics simulation

The simulation of CypA in the presence (holo) and absence (apo) of ligand was performed using the GROMACS 5.1.4 tool [38] as previously reported [37,39–41]. The topology of the CypA was created using GROMACS utilities, whereas the topology of the ligand was generated using the PRODRG server in the framework of the GROMOS96 43a1 force field [42,43]. All starting structures were solvated in a simple point charge water box under periodic boundary conditions using a 1.0 nm distance from the protein to the box faces [44]. Both apo and holo systems were then neutralized by 2 Cl^−^ ions. Following steepest descent energy minimization, the systems were equilibrated under NVT (constant number of particles, volume, and temperature) conditions for 60 ps at 300 K, followed by 60 ps under NPT (constant number of particles, pressure, and temperature) conditions. Temperature was maintained using the velocity-rescaling thermostat (modified Berendsen algorithm) with a coupling constant of 0.02 ps (nsttcouple = 10, dt = 2 fs) at a reference temperature of 300 K. The pressure was controlled using the Parrinello-Rahman barostat with isotropic coupling and a coupling time constant of 2 ps at a reference pressure of 1 bar [45,46]. The velocity-rescaling thermostat is known to produce a correct canonical ensemble by including a stochastic term that properly samples the kinetic energy distribution. Parinello–Rahman pressure coupling allows the simulation box to fluctuate in size appropriate to the isothermal–isobaric ensemble. The electrostatic interactions were treated using the Particle Mesh Ewald (PME) method [47,48] for evaluating electrostatic energies and forces of large periodic systems. Finally, a 100 ns MD was performed to analyze the stability of each system. MD simulation was all done on a Core i7 system containing an NVidia GeForce Graphical Processing Unit (GPU).

### Molecular dynamics trajectory analysis

#### Trajectory processing and analysis framework.

All MD trajectories were analyzed using GROMACS modules with a pipeline (see Supplementary S1 Data) implemented to compare the holo form (CypA in complex with the SangfA) and the apo form (unliganded CypA). Trajectories were centered prior to analysis to remove global translation. An index file was used for the holo system, containing pre-defined groups for protein, ligand, and solvent; for the apo system, default index groups were generated using *gmx make_ndx*.

#### Conformational stability and fluctuation metrics.

Root-mean-square deviation (RMSD) of Cα and backbone atoms was calculated relative to the initial frame, with time reported in nanoseconds. Per-residue root-mean-square fluctuation (RMSF) was computed for Cα atoms to quantify local flexibility. The solvent-accessible surface area (SASA) was determined for the entire protein using a probe radius of 0.14 nm. RMSD, RMSF, and SASA were measured by GROMACS’s *gmx rms*, *gmx rmsf*, and *gmx sasa* modules.

#### Hydrogen bonding and non-covalent interactions.

Hydrogen bonds were identified using a donor-acceptor distance cutoff of 0.35 nm and an angle cutoff of 30°. Three classes were analyzed for the holo system: protein–ligand, protein–water, and ligand-water. For the apo system, protein-water hydrogen bonds were quantified. Salt bridges were analyzed using *gmx saltbr* with a distance cutoff of 0.5 nm and were filtered to exclude interactions involving solvent or ligand. Protein-ligand electrostatic interactions were further probed by calculating minimum distances between charged residues, including ARG, LYS, HIS, GLU, ASP and the ligand using *gmx select* with a 0.5 nm threshold.

#### Essential dynamics and conformational sampling.

Principal component analysis (PCA) was performed on Cα atoms after least-squares fitting to the reference structure. Covariance matrices were built over the 20–100 ns period by *gmx trjconv*, and the first four eigenvectors were projected to examine concerted motions. The covariance matrix of atomic fluctuations was constructed for the C-alpha atoms, and eigenvalues and eigenvectors were obtained using *gmx covar* and *gmx anaeig*. The first four principal components (PCs) with the largest eigenvalues (83.42%) were computed based on the following equations to capture the most significant conformational changes. Equation 1 calculates the total variance of the system as the sum of all eigenvalues, providing a normalization factor for subsequent calculations. Equation 2 determines the fraction of total motion captured by each principal component, allowing us to identify which collective modes contribute most significantly to the protein’s conformational dynamics. Equation 3 accumulates the explained variance across successive principal components, enabling assessment of how many modes are required to describe a given percentage of the total variance of motion.


$$\begin{array}{c} \text{Total Variance}=\sum_{i=\text{1}}^{\text{N}}\lambda_{i} \end{array}$$ (1)

where λ_*i*_ is the *i*th eigenvalue.


$$\begin{array}{c} \text{Explained Variance }(\%)=\frac{\lambda_{i}}{Totalvariance}\times \text{1}\text{0}\text{0} \end{array}$$ (2)

$$\begin{array}{c} {CumulativeVariance}_{i}=\sum_{j}^{i}{ExplainedVariance}_{j} \end{array}$$ (3)

The projections of the atomic fluctuations onto these PCs were visualized to interpret the essential dynamics of the protein-ligand complex. Conformational clustering was carried out using the GROMOS method with an RMSD cutoff of 0.10 nm on Cα atoms, generating representative structures and population distributions.

#### Secondary structure, contact analysis, and torsional dynamics.

Secondary structure evolution was assessed using the DSSP algorithm. Secondary structure compositions derived from DSSP assignments were compared between apo and holo systems using a chi‑square test of homogeneity on the full 8‑state count table (H, B, E, G, I, T, S, ~) as described before [40]. Residue-residue contact maps were generated with *gmx mdmat* using a cutoff of 0.50 nm. For the holo system, binding pocket contacts were specifically evaluated by defining two loop regions, residues 50–70 and 100–120, and calculating contact frequencies within 0.45 nm of the ligand. Backbone dihedral distributions were analyzed via Ramachandran plots using *gmx rama* to assess allowed conformational states.

#### Calculation of linear interaction energy (LIE).

The binding free energy of the CypA-SangfA complex was estimated using the LIE method [49]. The LIE method relates the binding free energy to the difference in average interaction energies of the ligand with its surrounding environment in the bound and unbound states. According to the LIE formalism [49], the binding free energy is given by:


$$\begin{array}{c} LIE=\alpha \left({\Delta \text{G}}_{LJ,bound-unbound}\right)+\beta \left({\Delta \text{G}}_{Coul,bound-unbound}\right) \end{array}$$ (4)

$$\begin{array}{c} {\Delta \text{G}}_{\text{bind}}=\alpha \left({\left\langle V_{LJ,lig-surr}\right\rangle }_{bound}-{\left\langle V_{LJ,lig-surr}\right\rangle }_{unbound}\right)+\beta \left({\left\langle V_{Coul,lig-surr}\right\rangle }_{bound}-{\left\langle V_{Coul,lig-surr}\right\rangle }_{unbound}\right) \end{array}$$ (5)

*α* and *β* are LIE parameters.

${\left\langle V_{vdw,lig-surr}\right\rangle }_{bound}$ and ${\left\langle V_{vdw,lig-surr}\right\rangle }_{unbound}$ represent the average van der Waals and electrostatic interaction energies between the ligand and its surrounding environment (protein and solvent), respectively. The subscripts ‘bound’ and ‘unbound’ refer to the ligand in complex with the protein and the ligand free in solution, respectively. The parameters α and β are empirical scaling coefficients that account for the different contributions of the interaction energies to the binding free energy. Following established LIE protocols [49], the coefficients were set to α = 0.18 and β = 0.50. These values have been widely validated for protein-ligand binding affinity predictions [49].

The average interaction energies were extracted from the last 80 ns of the MD trajectories (20–100 ns) after equilibration, using the GROMACS *gmx energy* module. The simulations for the unbound state were performed by simulating the ligand alone in a water box under identical conditions as the bound-state simulations.

Also, ${\left\langle V_{ele,lig-surr}\right\rangle }_{bound}$ and ${\left\langle V_{ele,lig-surr}\right\rangle }_{unbound}$ are the differences in average van der Waals and electrostatic interaction energies between the bound and unbound states.

### Statistical analysis

All statistical analyses were performed using R v4.5.2 RMSD, RMSF, SASA, and H-bonding distributions were characterized by mean, median, standard deviation, and interquartile range. Per‑residue probabilities of α-helix (H+G+I) and β-strand (E+B) were assessed for significant differences using two‑tailed Student’s t‑tests and Mann-Whitney U tests, with multiple‑testing correction applied via the Benjamini-Hochberg procedure (false discovery rate = 0.05). Effect sizes were quantified as Cohen’s d (pooled standard deviation). PCA of Cα atomic fluctuations was conducted after superposition onto a reference structure, initial frame, and diagonalization of the covariance matrix; eigenvalues and cumulative variance contributions were computed to assess the dimensionality and collectivity of conformational motions. Conformational space volumes were estimated as the product of the standard deviations along PC1 and PC2. The number of effective clusters’ conformations was calculated as $Exp\left(\text{1}\sum p_{i}\text{ln}p_{i}\right)$, where *p_i_* is the fractional population of cluster *i*. Ramachandran φ/Ψ angles were assigned to sterically favored, allowed, generous, and outlier regions according to literature definitions; the fraction of outliers was compared qualitatively. All temporal traces were smoothed with a 100‑frame moving average equivalent to 1.25 ns to facilitate visualization of low‑frequency trends. Statistical significance was defined as p < 0.05 unless otherwise noted.

## Results

### Conformational Stability of AypA in Apo and Holo States

**Fig 1:**
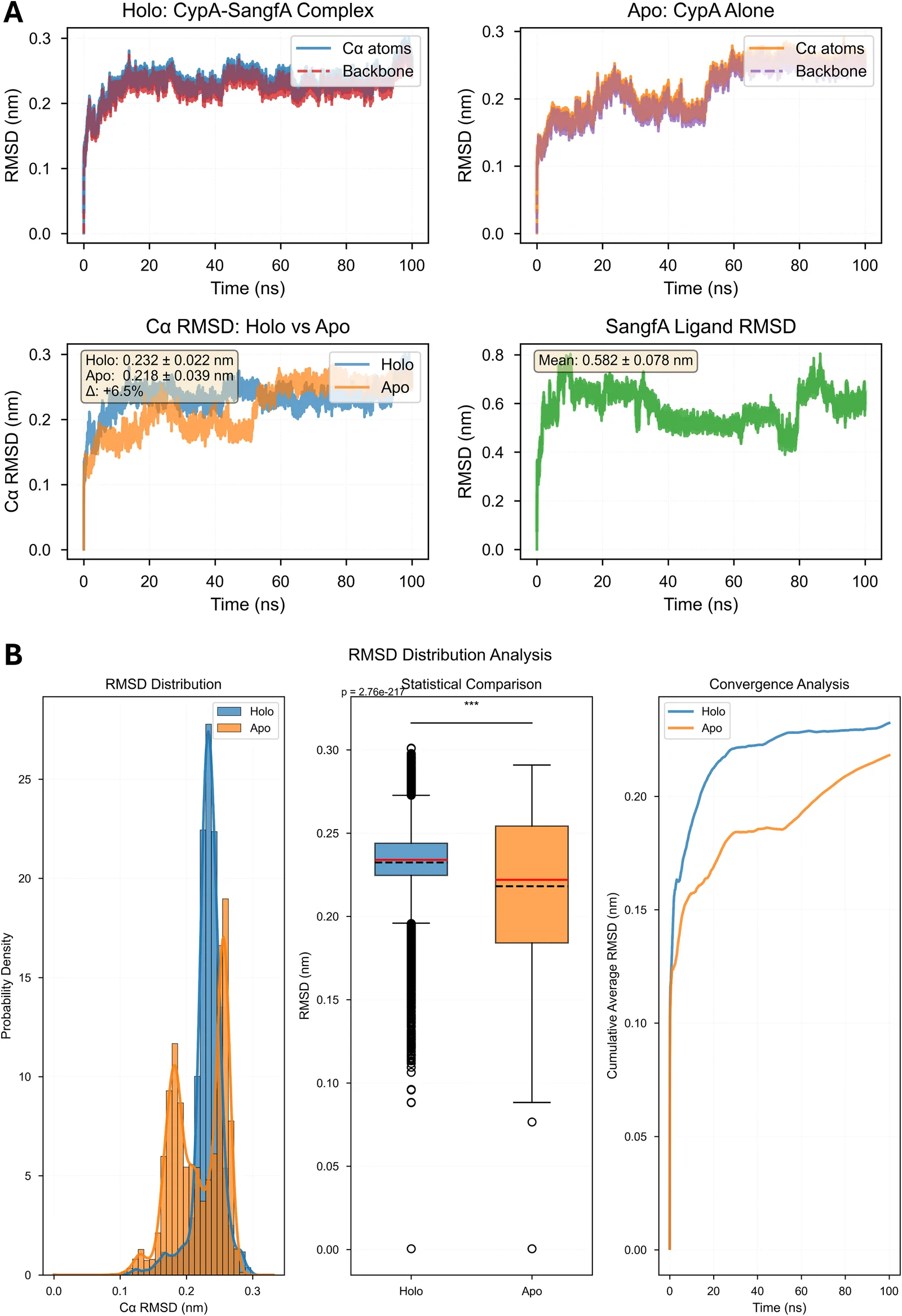
Time series of RMSD for the apo and holo simulations. A) Solid lines show Cα RMSD; dashed lines show full backbone RMSD. Inset: corresponding ligand RMSD (green) after fitting on the protein. The ligand-bound form exhibits a higher mean RMSD but much narrower fluctuations than apo. B) Histograms of backbone RMSD for apo and SangfA-bound CypA. The bound complex has a significant higher average RMSD and a narrower distribution, indicating reduced dynamic heterogeneity upon binding.

RMSD analysis of the protein backbone and C_α_ atoms revealed that both the apo and holo systems achieved stable conformational equilibria following an initial equilibration phase (Fig 1). The holo complex (Cα RMSD: 0.232 ± 0.022 nm; backbone RMSD: 0.224 ± 0.021 nm) exhibited higher overall deviation than the apo form (Cα RMSD: 0.218 ± 0.039 nm; backbone RMSD: 0.212 ± 0.038 nm), although the apo system demonstrated a broader distribution (IQR: 0.070 nm) compared to the holo form (IQR: 0.019 nm). This suggests the ligand introduces a subtle constraint, reducing conformational heterogeneity. The SangfA itself maintained a stable binding pose with an average RMSD of 0.582 ± 0.078 nm relative to the protein-fitted reference.

### Ligand binding modulates local protein flexibility

**Fig 2:**
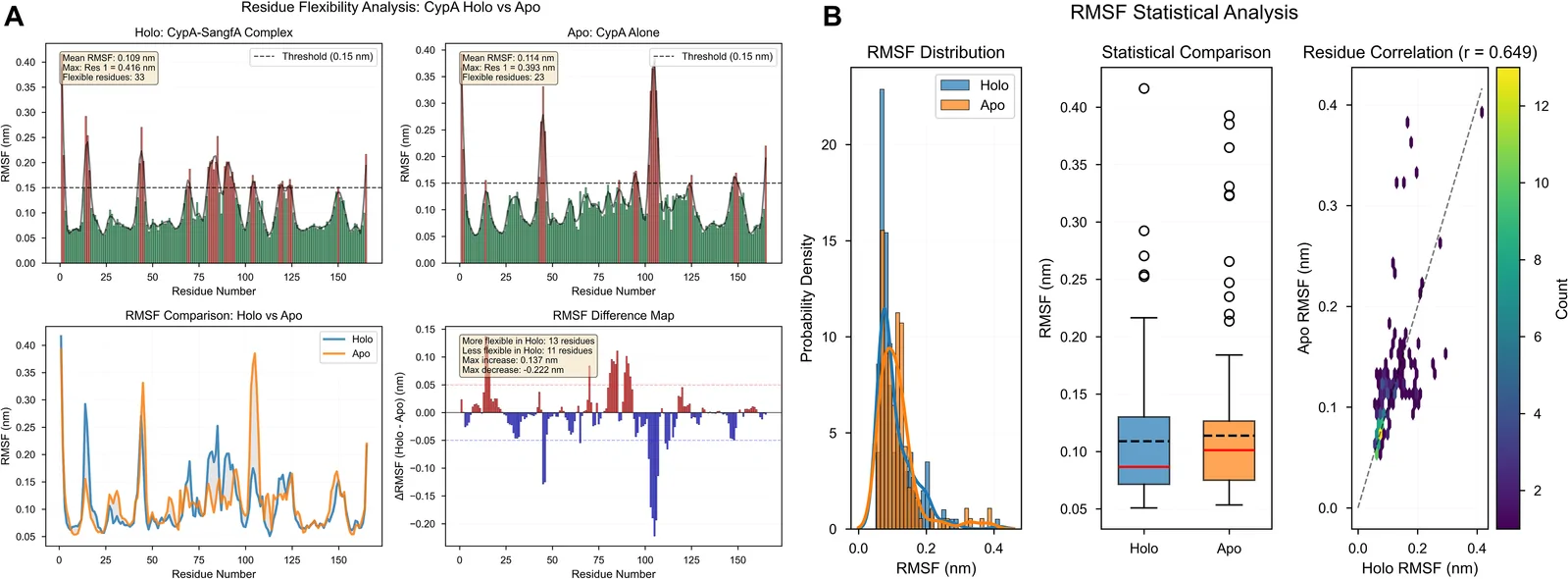
Per-residue RMSF of Cα atoms for apo and SangfA-bound CypA. A) Arrowheads mark residues showing marked stabilization upon ligand binding; arrows mark residues with increased flexibility. The most flexible segments N-terminus and C-terminal tail are restrained by binding, while certain loops residues 13–17, 69–73 become more mobile. B) statistical analyzes of RMSF fluctuation. The fluctuation of residues 100–110 are significantly repressed upon SangfA binding. A significant correlation was also observed between CypA residues’ RMSF in apo and holo systems.

Per-residue RMSF of Cα atoms demonstrated the effect of ligand binding on local dynamics (Fig 2). The mean residue fluctuation decreased slightly upon ligand binding (holo: 0.109 ± 0.054 nm; apo: 0.114 ± 0.060 nm). A more pronounced effect was observed in the distribution of high-flexibility residues (defined as RMSF > mean + 1σ). While both systems contained 17 such residues, the flexibility threshold was higher in the holo state (0.187 nm) than in the apo state (0.162 nm).

#### Ligand binding induces a conformational adjustment with increased solvent exposure.

**Fig 3:**
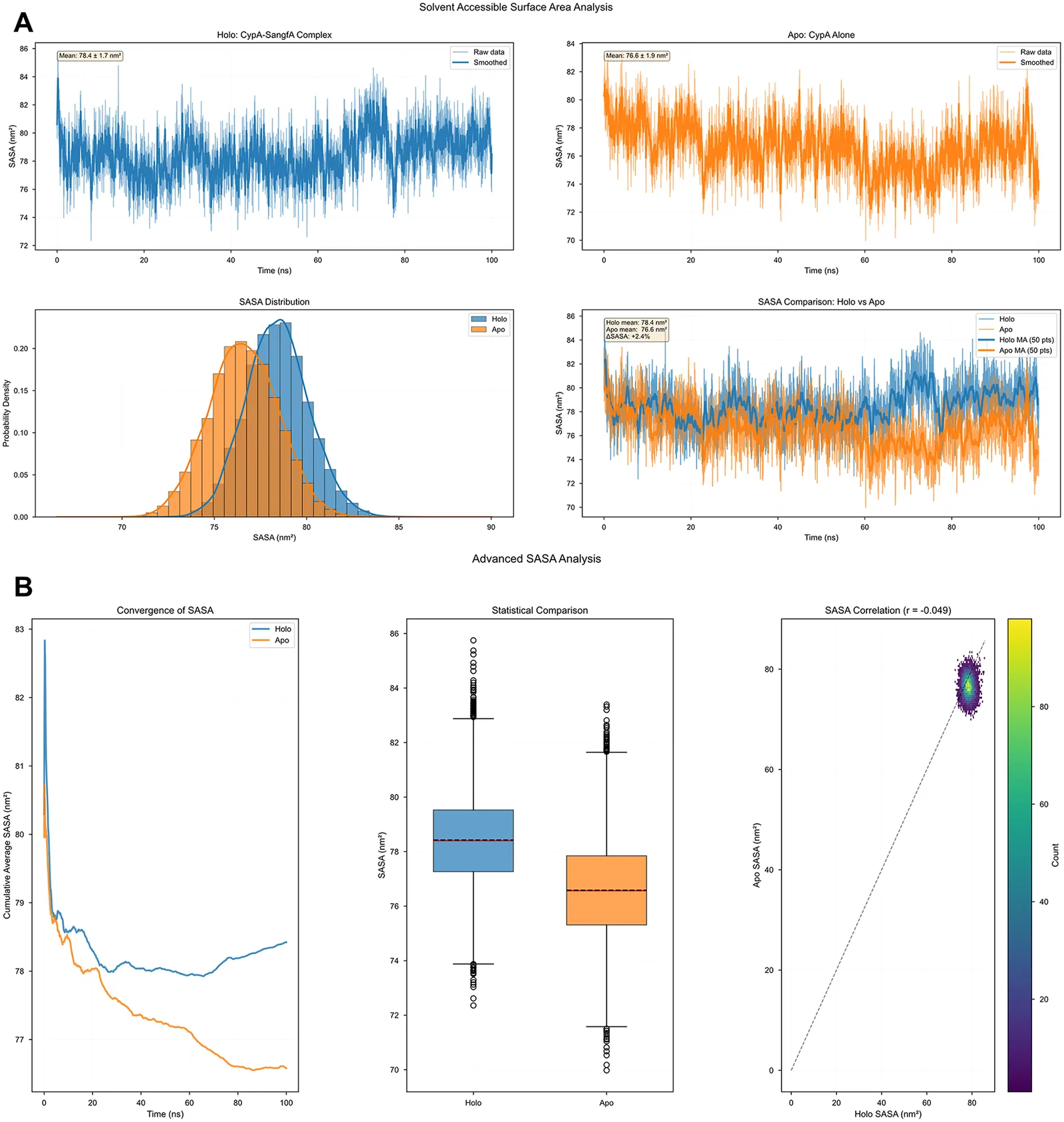
SASA analysis of CypA ensembles. Time evolution of the solvent accessible surface area for (A) the apo form of CypA and (B) the holo. (C) Probability distribution of the SASA values for both systems over the simulation trajectory. The data illustrate the impact of ligand binding on the overall solvent exposure of the protein, indicating potential compaction or stabilization of the hydrophobic core upon complex formation.

Analysis of the total SASA revealed a consistent increase in the holo system (78.42 ± 1.69 nm^2^) compared to the apo form (76.58 ± 1.86 nm^2^) (Fig 3). This 2.4% expansion in accessible surface area, significant at p < 0.001 (two-sample t-test), suggests the bound ligand induces a conformational adjustment that increases the protein’s overall solvent exposure, potentially through slight rearrangements of peripheral loops or side chains.

### Hydrogen-bonding network reveals stable hydration and dynamic ligand interactions

**Fig 4:**
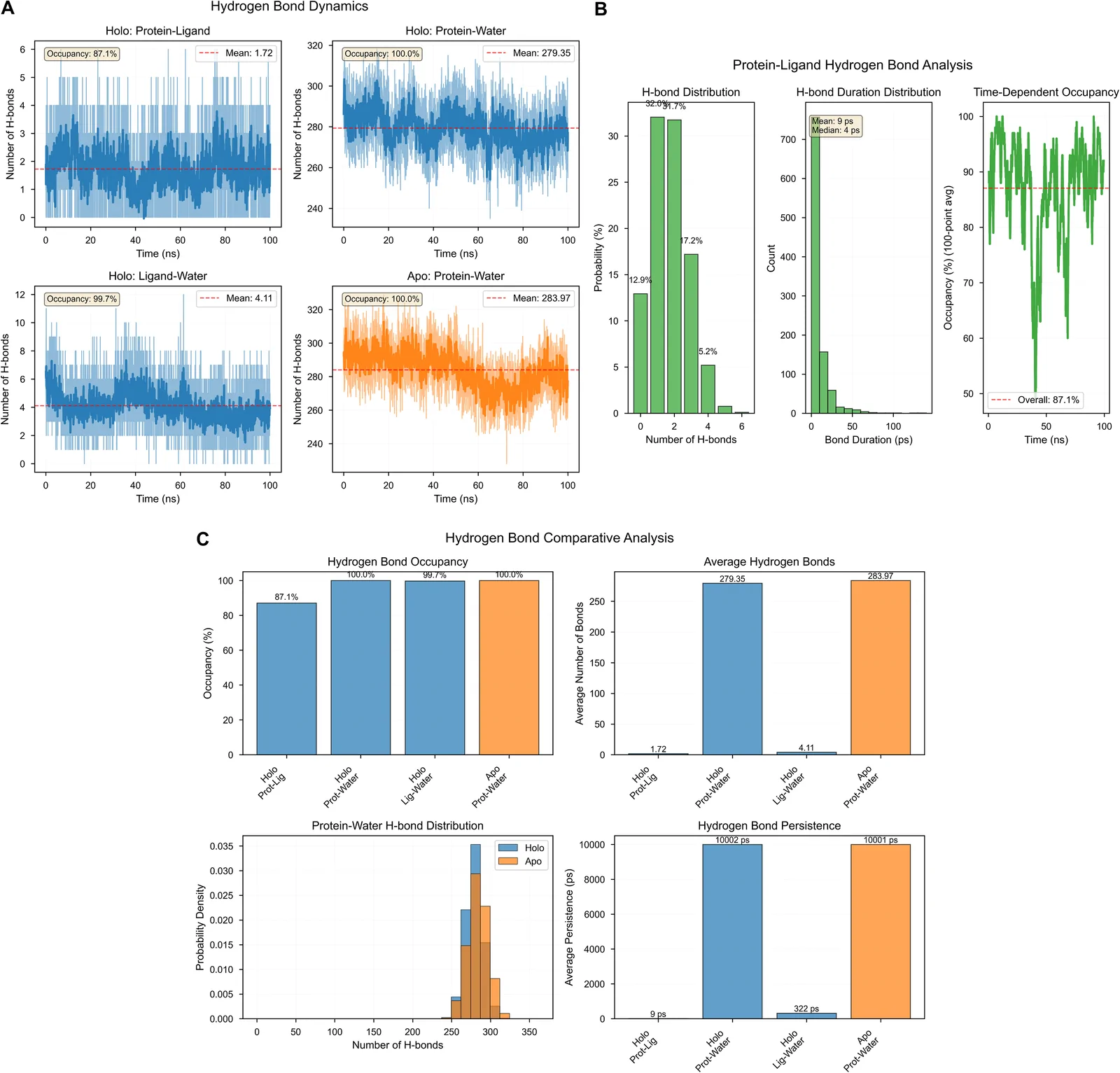
Intermolecular hydrogen bonding analysis. A) Total number of intermolecular hydrogen bonds formed between the CypA-water and CypA-SangfA. Results demonstrate reduced CypA-water hydrogen binding upon SangfA bonding from 284 to 279. Also, SangfA has hydrogen bonding with both water and CypA, highlighting its stable RMSD fluctuation. B) Bar charts of hydrogen bonds alalyzes. H bonds were formed between CypA and SangfA in a range of 0% to 32% probabilities with a mean of 9 ps duration, and 87% occupancy. (C) Further detailes the hydrogen bonding dynamics in the systems.

Hydrogen bond analysis detailed the distinct interaction profiles of the two systems. The protein-water network was extensive and persistent in both states, with near-100% occupancy. The holo system maintained an average of 279.35 ± 10.80 protein-water H-bonds, slightly fewer than the apo state (283.97 ± 12.85), likely due to partial displacement of hydration shells in the binding pocket (Fig 4).

The protein-ligand interface was characterized by dynamic, transient hydrogen bonds. An average of 1.72 ± 1.11 bonds were present per frame, with an overall occupancy of 87.1%. When formed, these bonds had an average persistence time of only 8.5 ps, indicating a highly dynamic interaction mode rather than static, locked-in contacts. The ligand remained well-hydrated, maintaining 4.11 ± 1.56 stable H-bonds with solvent (99.7% occupancy), which may facilitate its binding and release kinetics.

#### The ligand binds in close proximity, anchored by a specific, persistent aspartate interaction.

**Fig 5:**
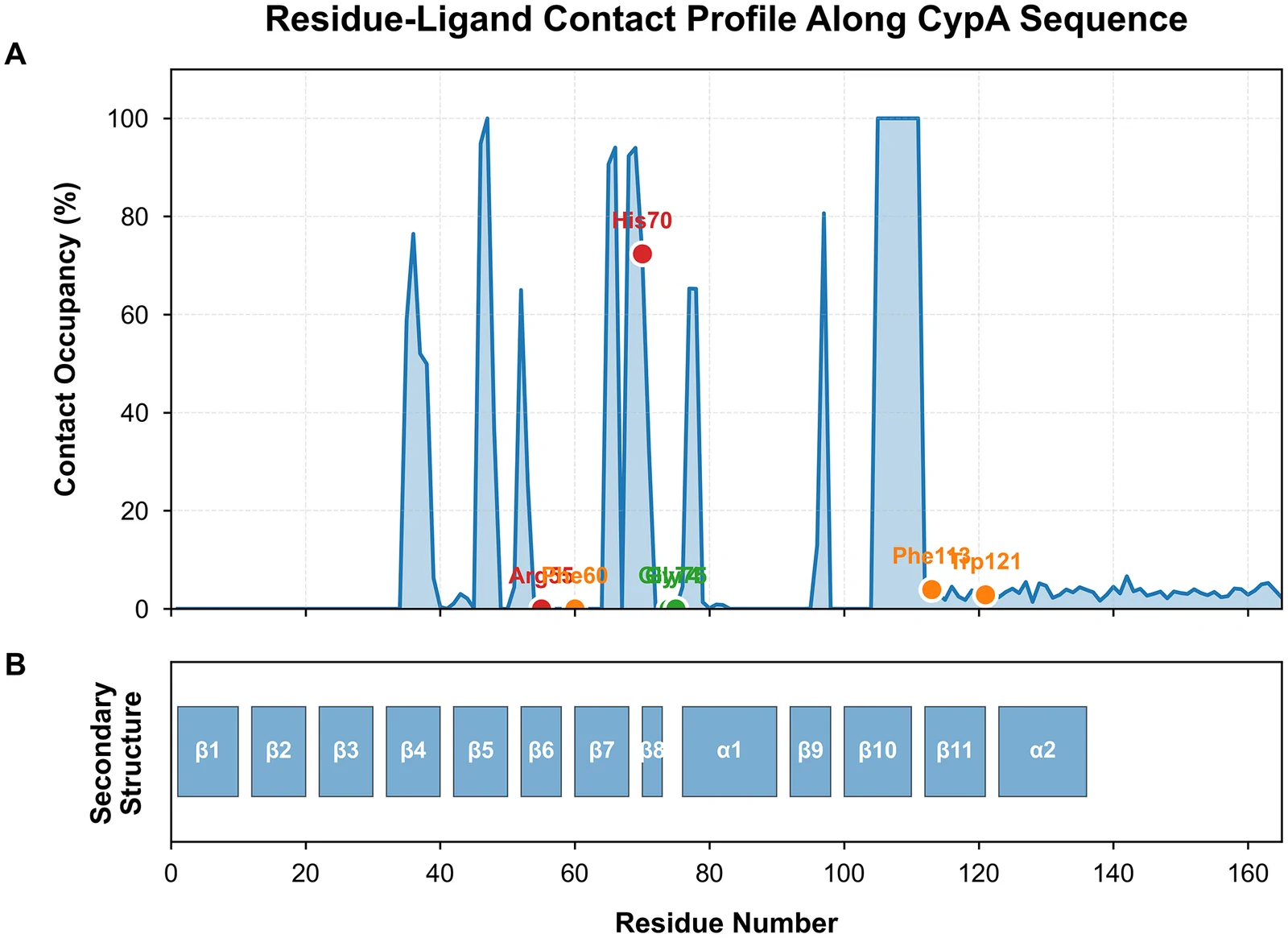
Per-residue interaction frequency between the SangfA ligand and CypA throughout the simulation. A) The profile identifies hotspots of interaction, with the active site residues His70 shows the highest contact occupancy, confirming the binding pocket architecture. B) A manually placed secondary structure of CypA relative to the close SangfA contact profile on CypA.

**Fig 6:**
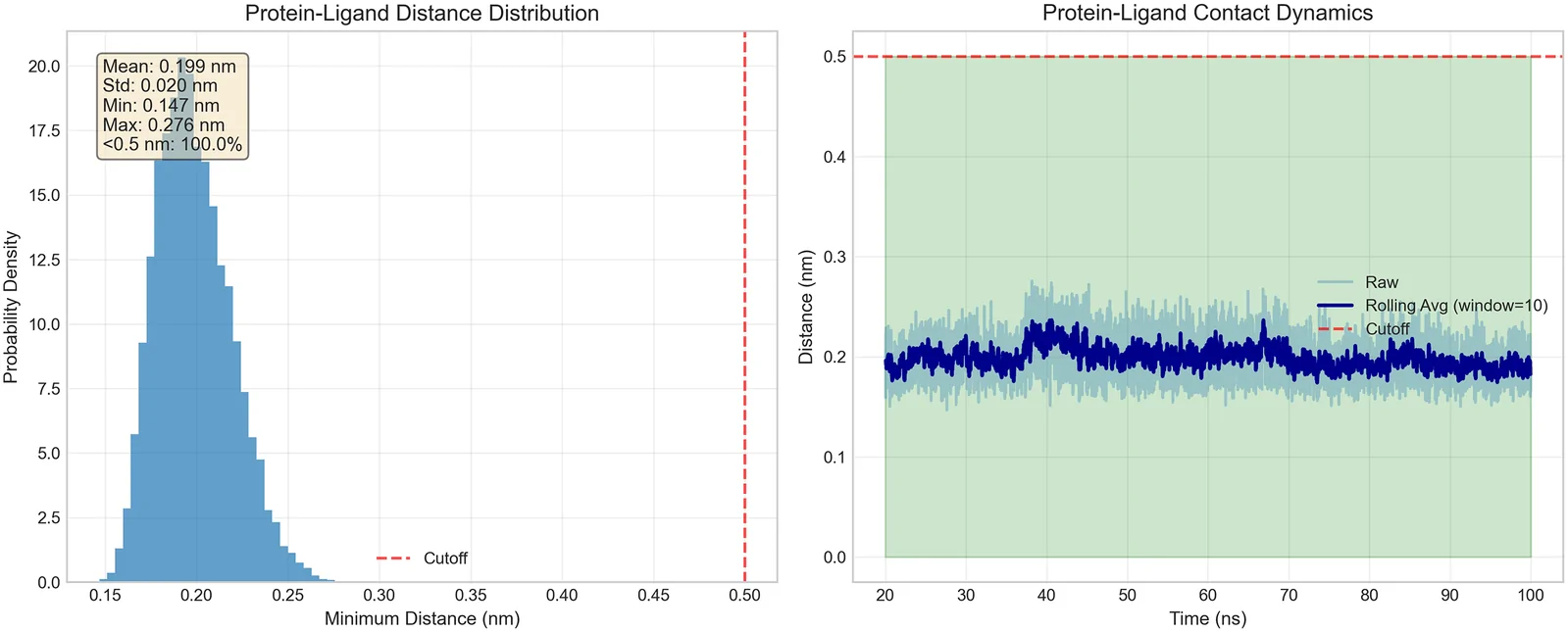
Minimum distance analysis between SangfA and CypA. Time evolution of the minimum distance between the SangfA ligand and the CypA binding site residues and the entire protein (right panel). Histogram distribution of these minimum distances (left panel), demonstrating high density of contacts sustained close (< 0.25 nm; right panel), indicative of a tightly bound complex without dissociation events. Cutoff = 0.5 nm.

Analysis of the minimum distance between the CypA and the SangfA revealed a stable, close binding interface (Fig 5). The average minimum distance was 0.199 ± 0.020 nm, with a range of 0.147–0.276 nm (Fig 6). This close proximity was maintained with 100% occupancy throughout the analyzed trajectory, indicating no dissociation events and confirming a stable bound state.

Residue-specific contact analysis of charged amino acids (Table 1), however, revealed a highly selective interaction profile. ASP residues demonstrated a dominant role in ligand coordination. The average distance for ASP contacts was short at 0.119 ± 0.408 nm, with a high occupancy of 90.4% for distances below the 0.5 nm cutoff. In contrast, contacts with other charged residues were infrequent or distant. Accordingly, LYS residues had an average distance of 8.63 nm and only 9.2% occupancy, GLU averaged 6.69 nm with 1.6% occupancy, and arginine ARG residues were never within the cutoff distance, averaging 16.67 nm away.

**Table 1:** Statistics for charged residue contacts with the Sanglifehrin A derivative ligand.

Residue Type	Avg. Distance (nm)	Occupancy (<0.5 nm)	Interaction Character
ASP	0.119 ± 0.408	90.4%	Persistent, Anchor
LYS	8.634 ± 4.968	9.2%	Transient
GLU	6.687 ± 2.529	1.6%	Negligible
ARG	16.670 ± 5.907	0.0%	Non-interacting

#### The binding interface is characterized by a specific ionic interaction rather than distributed salt bridges.

Despite the general lack of classical salt bridges, the protein-ligand interface features a persistent electrostatic interaction involving aspartate. The data indicate that one or a few specific ASP residues, including ASP-55, a known key residue in the CypA active site, form a stable, close-contact interaction with the ligand. This specific ionic or hydrogen-bonding interaction, with an average distance well below the standard salt bridge cutoff, appears to serve as a primary anchor point. The minimal involvement of other charged residues LYS, GLU, ARG suggests the binding affinity and specificity are driven by this focused electrostatic attraction, complemented by the dynamic hydrogen-bonding network and hydrophobic contacts described previously, rather than by multiple, distributed salt bridges.

#### The ligand binds within a defined, multi-residue interface centered on the β strand.

Residue-specific contact analysis identified a comprehensive binding interface between CypA and the SangfA, comprising 95 of 165 residues (57.6%) with non-zero contact occupancy. The interaction profile was highly heterogeneous, dominated by a cluster of five residues, including PHE102, GLU103, ALA104, LYS105, ALA106 that maintain 100% contact occupancy throughout the simulation, forming the invariant core of the binding site. An additional 12 residues exhibited high (50–90%) or very high (90–100%) occupancy, including PHE46 (β5, 100%), LYS64, LYS66, THR63 (β7, 90–94%), and LYS93 (β9, 80.6%). This pattern delineates a contiguous binding surface spanning several β-strands and the β8-α1 loop.

#### Chemical character of the interface reveals a predominance of basic residues and specific hydrophobic patches.

Analysis of the contacting residues by chemical type revealed distinct contributions to binding stability (Table 2). Basic residues Lys, Arg, His, though fewer in number, displayed the highest mean occupancy (22.28 ± 30.87%), driven by the persistent interactions of LYS105, LYS64, LYS66, and LYS93. In contrast, the more hydrophobic residues played a supportive role with lower average occupancy (10.24 ± 21.64%), except for the critical PHE102 and PHE46. Acidic residues had low mean occupancy (9.29 ± 26.10%), with the exception of GLU103. This profile suggests the binding affinity is significantly driven by electrostatic interactions with basic side chains, supplemented by key hydrophobic anchoring points.

**Table 2:** Summary of residue contact statistics by chemical type.

Residue type	No. of contacting residues	Mean occupancy ± SD (%)	Representative high-Occupancy residues (Occupancy %)
Basic	17	22.28 ± 30.87	LYS105 (100.0), LYS64 (94.0), LYS66 (92.3)
Hydrophobic	39	10.24 ± 21.64	PHE102 (100.0), PHE46 (100.0), ILE68 (72.4)
Polar	27	10.20 ± 19.68	THR63 (90.7), ASN36 (58.8), GLN45 (49.7)
Acidic	12	9.29 ± 26.10	GLU103 (100.0), GLU75 (65.3)

#### The catalytic site shows partial engagement while the canonical hydrophobic pocket is largely bypassed.

Comparison with known functional sites of CypA reveals a nuanced binding mode. The catalytic site residue LYS70 exhibited moderate engagement (32.5% occupancy), whereas the equally critical ARG55 showed no contact (0% occupancy). This suggests the ligand interacts with, but does not fully occupy or block, the enzymatic active site. Conversely, residues constituting the canonical hydrophobic substrate pocket PRO113, GLY60, GLU121 demonstrated negligible contact (occupancy ≤ 3.0%). Instead, the ligand forms its primary stabilizing interactions with the high-occupancy β cluster, which lies outside this pocket. The glycine loop (residues 74–75) shows mixed involvement, with GLU75 forming a frequent interaction (65.3% occupancy) while LYS74 is rarely contacted (4.5%).

### Ligand binding increases conformational diversity and reduces structural convergence

Clustering analysis of the Cα atoms with a 0.10 nm cutoff revealed a significant change in the conformational landscape upon ligand binding. The holo system populated a greater number of distinct conformational states (75 total clusters) compared to the apo system (66 total clusters). Moreover, the distribution of conformations was markedly different. Accordingly, the largest cluster in the apo state encompassed 40.0% of the trajectory frames, 3,200 of 8,001 frames, indicating a dominant, stable conformation. In contrast, the largest cluster in the holo state contained only 12.2% of frames, 976 of 8,002 frames. Consequently, the number of effective conformations, calculated as total frames divided by the average cluster size, increased by 124% upon ligand binding (Holo: 75.0; Apo: 66.0).

**Fig 7:**
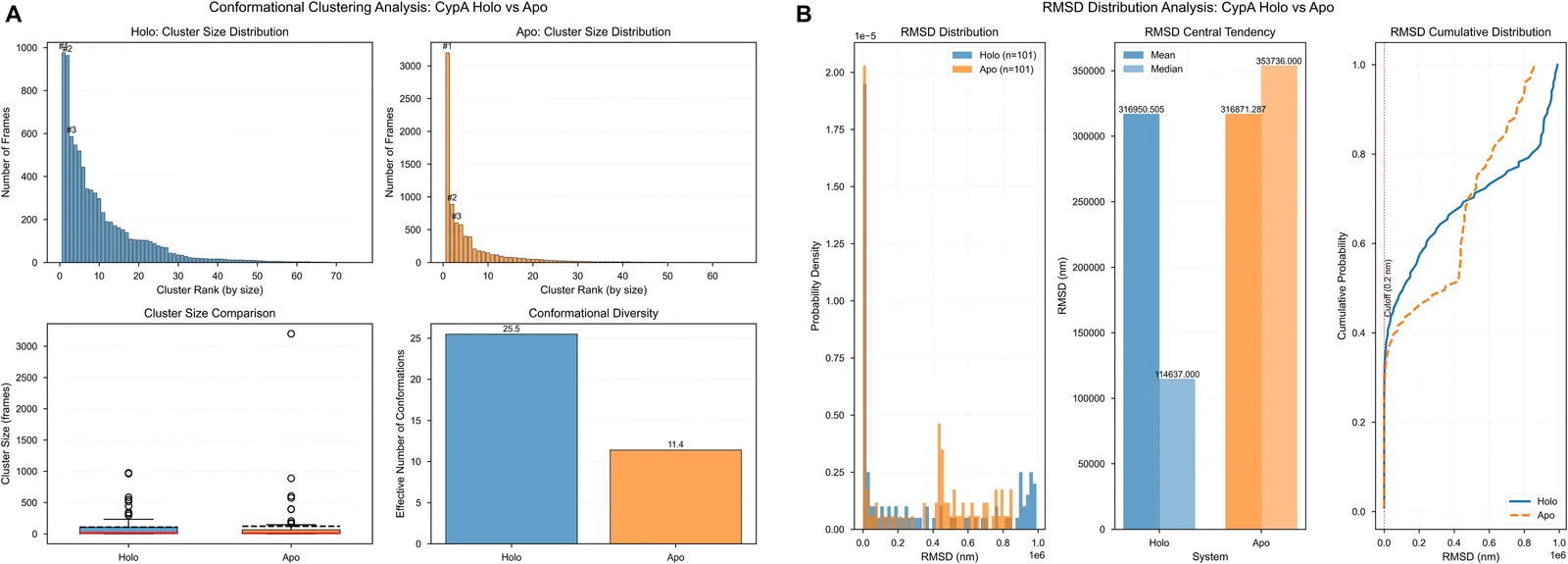
Cluster analysis of conformational ensembles. (A) Clustering was performed using the GROMOS algorithm with an RMSD cutoff of 0.1 nm on Cα atoms. A) The largest cluster in holo contains only 976 frames, whereas the apo ensemble is dominated by a single cluster comprising 3,200 frames, reflecting increased conformational heterogeneity upon SangfA bonding. The number of effective conformations (exp(Shannon entropy)) rises from 11.4 in apo to 25.5 in holo (+123.6%), consistent with a conformational selection mechanism. Singleton clusters were comparable (7 in holo, 9 in apo). (B) Pairwise RMSD distributions (Cα atoms, all-vs-all) exhibit distinct profiles. Mean RMSD values were nearly identical (0.317 nm for both systems), but the median RMSD is significantly lower in holo (0.115 nm) than in apo (0.354 nm), while the standard deviation is substantially higher in holo (0.375 nm vs. 0.298 nm in apo). The maximum RMSD reaches 0.990 nm in holo versus 0.854 nm in apo. The clustering and RMSD metrics demonstrated that SangfA expands the accessible conformational space and increases structural diversity, likely facilitating the recognition of diverse substrates.

Analysis of RMSD between all sampled cluster centroids provided a measure of the geometric diversity within each conformational ensemble (Fig 7). Both systems exhibited very large inter-cluster RMSD values, clustering captured highly disparate structural states, potentially including folded, partially unfolded, or significantly displaced loop conformations sampled over the 100 ns trajectory. While the mean RMSD was similar between states, the holo system displayed a 26% greater standard deviation (Holo: 374,970 nm; Apo: 298,466 nm) and a wider range. This greater dispersion in the holo state reinforces the conclusion from clustering that ligand binding increases the structural heterogeneity of CypA, broadening the distribution of accessible conformations rather than narrowing it.

**Fig 8:**
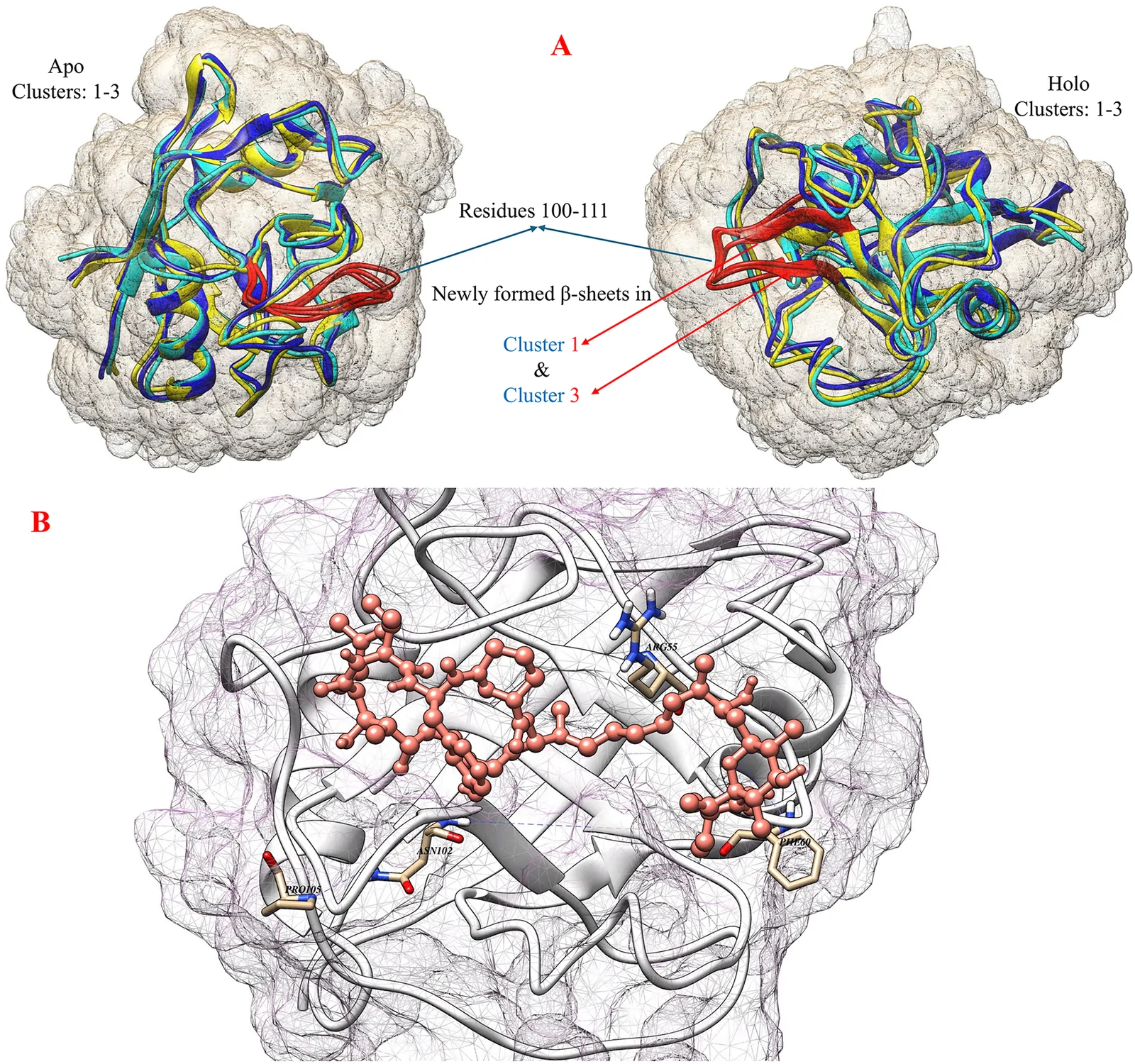
Representative structures from the MD simulations. (A) Superimposition of the top three most populated clusters from the apo and holo simulations (shown in different colors: blue, cyan, and yellow), with the β2–β3 loop region (residues 100–111) highlighted in red. Interesting secondary structure changes was observed in cluster 1 and cluster 3. Accordingly, Ile97, Leu98, Ser99, Met100, Ala101, and Asn102 were transformed from coil in apo to β-strand in holo. In cluster 3, however, only three Ile97, Leu98, and Ser99 amino acids were transformed from coil to β-strand (B) Close-up view of the binding pocket in the holo representative structure showing key interacting residues Arg55, Phe60, Phe102, Lys105, and the ligand SangfA.

**Fig 9:**
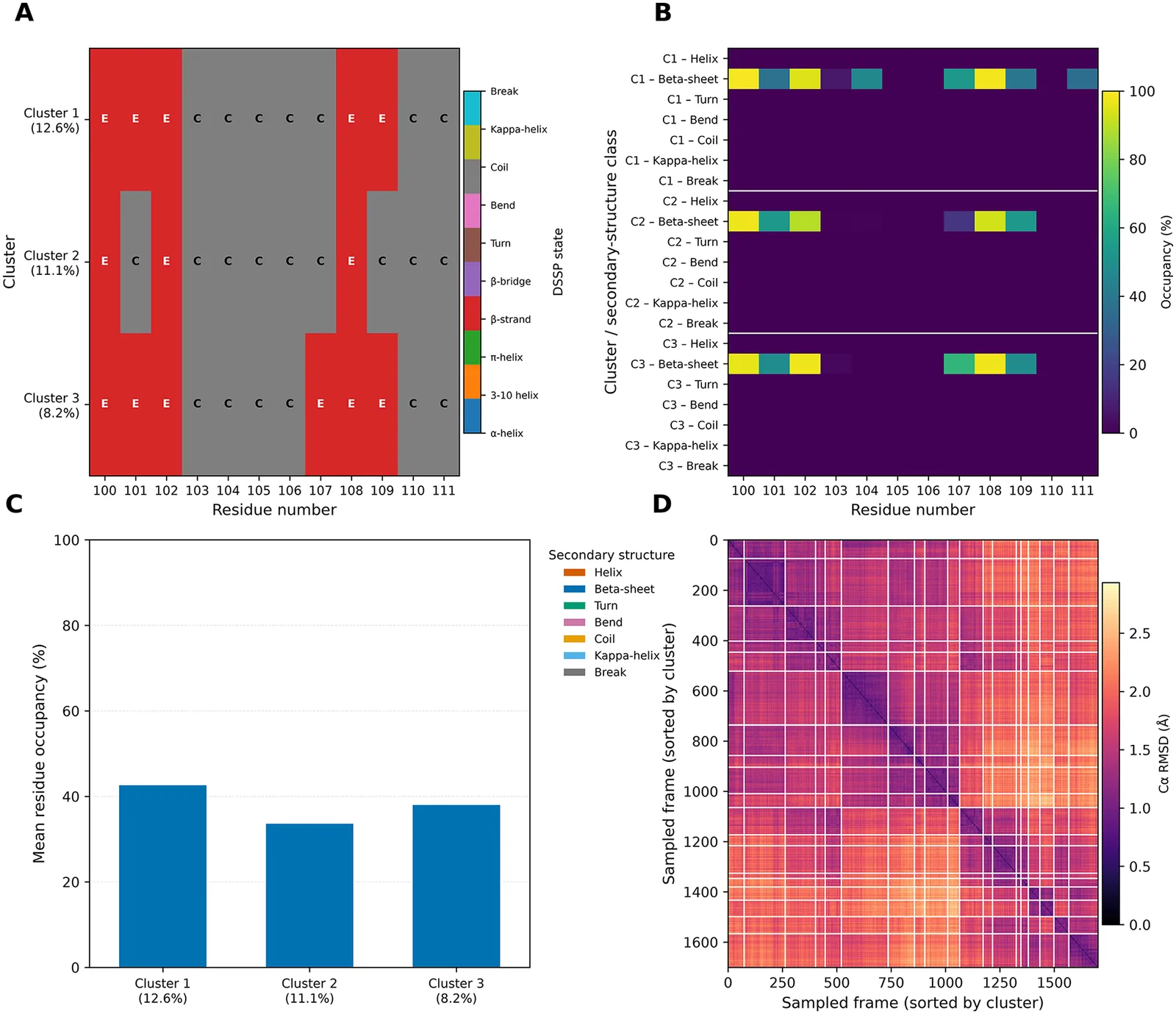
Conformational clustering and secondary-structure analysis of the holo CypA-SangfA system. (A) DSSP secondary-structure assignment across residues 100–111 for the three most populated conformational clusters. Each cell represents the DSSP code observed at the cluster medoid. Cluster populations are indicated in parentheses. DSSP codes: H (α-helix), G (3-10 helix), I (π-helix), E (β-strand), B (β-bridge), T (turn), S (bend), C (coil), P (kappa-helix), = (break). (B) Residue-level secondary-structure occupancy heatmap for residues 100–111. Occupancy percentages were calculated from all sampled frames belonging to each cluster. The heatmap displays the percentage of frames in which each broad secondary-structure category is observed at each residue position. Color intensity scales from 0% (dark blue) to 100% (yellow). (C) Secondary-structure composition of the 100–111 region across the three clusters. Stacked bar chart showing the mean residue occupancy of each secondary-structure class, calculated as the average across residues 100–111 for all frames within each cluster. Cluster populations are shown below each bar. β-strand content is the dominant structural feature in all three clusters, ranging from 16.84% (Cluster 2) to 21.34% (Cluster 1). (D) Cα RMSD distance matrix of the clustered trajectory frames. The matrix displays pairwise Cα RMSD values (Å) for all 1,701 sampled frames, sorted by cluster assignment. White horizontal and vertical lines indicate cluster boundaries. The hierarchical clustering at a 1.0 Å cutoff produced 20 clusters, with the three most populated clusters 1–3 shown in the ordered matrix. The color scale ranges from 0 Å (dark) to 2.93 Å (light), with intra-cluster RMSD values typically below 0.9 Å.

We have further performed a hierarchical clustering of the CypA-SangfA trajectory based on Cα RMSD to investigate the structural changes of the β2-β3 loop region residues 100–111 (Fig 8). The findings identified 20 distinct conformational clusters at a 1.0 Å cutoff, with the three most populated clusters comprising 12.58%, 11.11%, and 8.23% of the 1,701 sampled frames, respectively (Fig 9). Cluster 1 (n = 214 frames) spans the 31.4–42.1 ns time window with a medoid mean intra-cluster RMSD of 0.856 Å, Cluster 2 (n = 189 frames) occupies the 45.8–55.4 ns region with a medoid RMSD of 0.809 Å, and Cluster 3 (n = 140 frames) covers 55.15–62.25 ns with a medoid RMSD of 0.899 Å. The representative medoid structures for clusters 1–3 correspond to trajectory frames at 39.1 ns, 51.75 ns, and 59.85 ns, respectively. DSSP secondary-structure analysis of residues 100–111 at each cluster medoid revealed that β-strand content predominates in all three clusters, with cluster 1 showing β-strand (E) assignments at residues 100–102 and 108–109; cluster 2 at residues 100, 102, and 108; and cluster 3 at residues 100–102 and 107–109 (Figs 8A and 9). Residue-level occupancy analysis across all frames within each cluster demonstrated that the mean β-sheet occupancy for the 100–111 region was 21.34%, 16.84%, and 19.05% for clusters 1, 2, and 3, respectively. No significant helix, turn, bend, coil, kappa-helix, or break occupancy was observed in any of the three clusters, indicating that the 100–111 region predominantly samples β-strand conformations across all major conformational states upon bindings SangfA. The Cα RMSD distance matrix revealed distinct cluster separation, with intra-cluster RMSD values generally below 0.9 Å, while inter-cluster RMSD values extended up to 2.93 Å.

### Ligand binding induces subtle shifts in backbone dihedral distributions and secondary structure propensity

**Fig 10:**
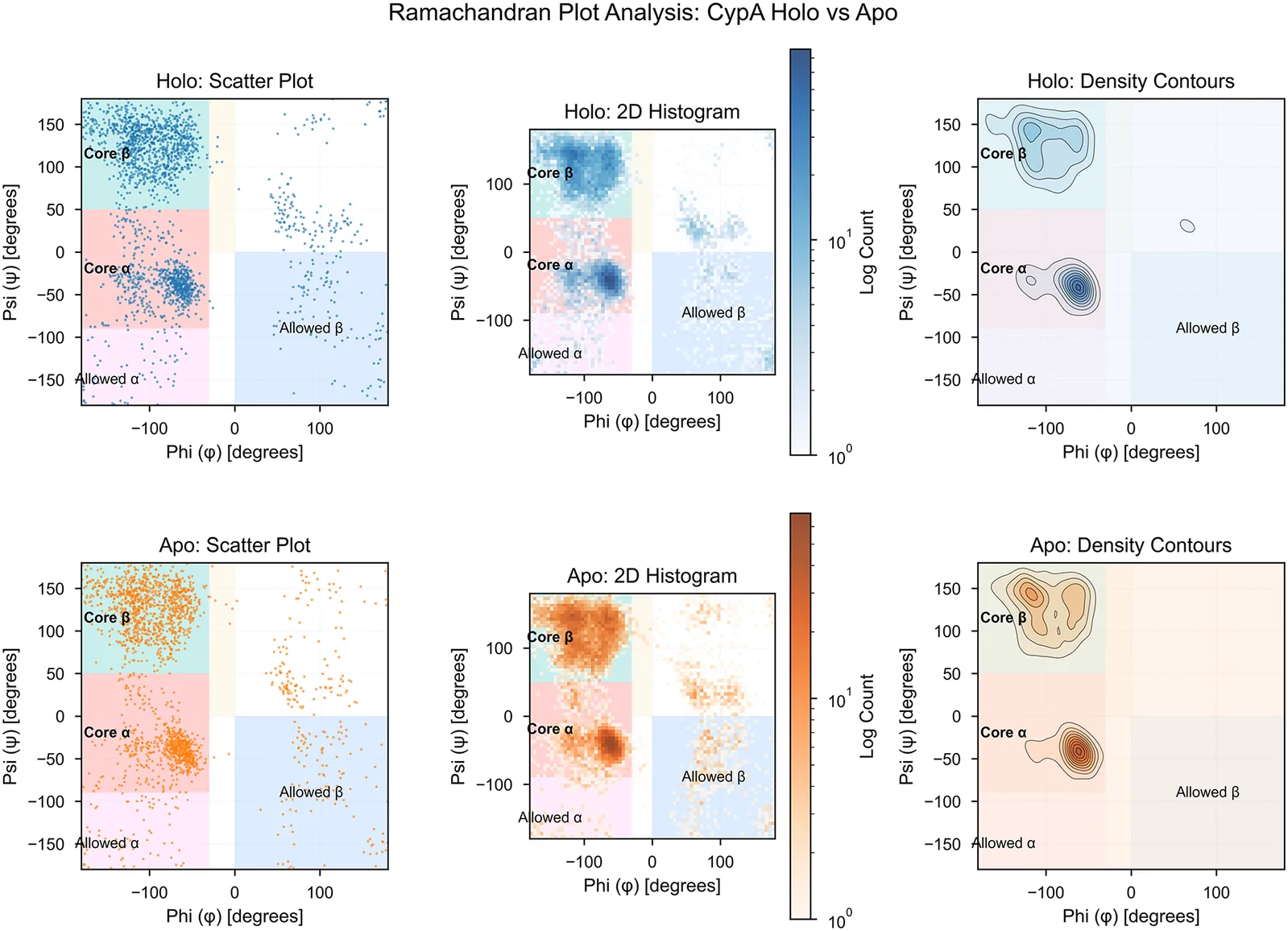
Conformational distribution of backbone dihedral angles (φ,ψ) for all non-glycine, non-proline residues. Ramachandran maps are contoured by density, with sterically favored regions (core α + core β) indicated by solid outlines and additionally allowed regions by dashed outlines. Secondary structure composition derived from φ/ψ occupancy: holo exhibits slightly higher α-helix content and marginally lower β-strand content relative to apo, suggesting a modest ligand-induced shift in backbone conformation. Fraction of residues in favored, allowed, generous, and outlier regions. Both systems show comparable stereochemical quality, with 83.6% (holo) and 83.8% (apo) of residues in favored regions. However, outlier populations remain considerable (6.2% in holo, 7.0% in apo). This may indicate local backbone strain of fully relaxed conformations. Mean φ angles were similar (−69.0° in holo, −67.8° in apo), while mean ψ angles differ more notably (44.2° in holo, 50.4° in apo), reflecting the altered secondary structure propensities.

Analysis of backbone φ and ψ dihedral angles via Ramachandran plots (Fig 10) revealed distinct conformational preferences between the apo and holo states of CypA (Table 3). The proportion of residues in the core α-helical region increased by 1.9 percentage points upon ligand binding. Concurrently, the population of the core β-strand region decreased by 2.1% points. The total percentage of residues in sterically favored regions remained high and stable in both systems.

**Table 3:** Summary of Ramachandran plot analysis for apo and holo CypA.

Region	Holo (%)	Apo (%)	Δ (Holo-Apo)
Core α-helix	33.53	31.60	1.93
Core β-strand	50.08	52.18	−2.10
Allowed	9.95	8.77	1.18
Generous	0.24	0.48	−0.24
Outlier	6.20	6.97	−0.77
Total Favored	83.61	83.78	−0.17

### The holo state exhibits a slight reduction in steric outliers and altered angular distributions

**Fig 11:**
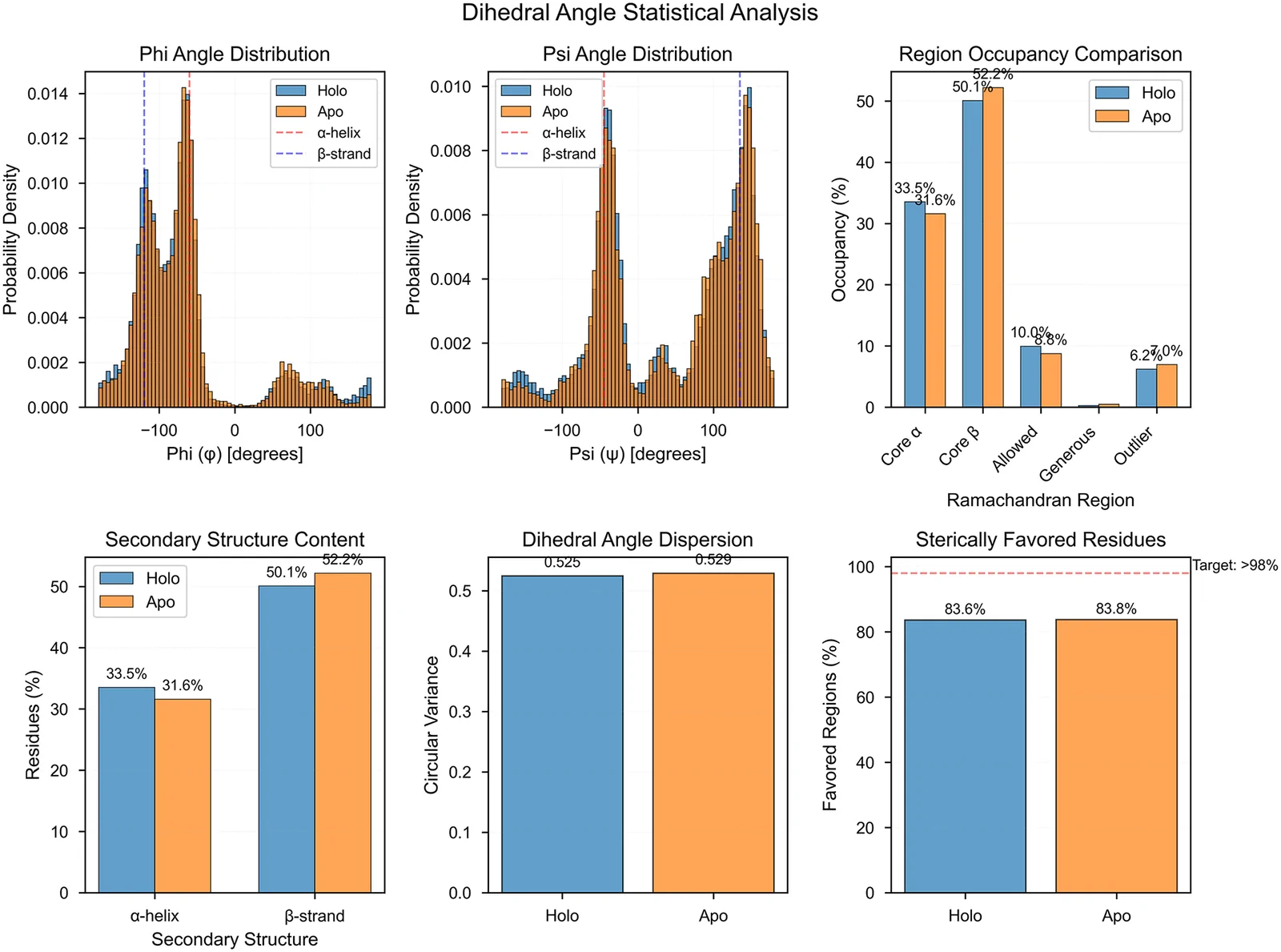
Dihedral angle statistical analysis. Probability density distributions of backbone φ and ψ angles for apo and holo CypA are depicted at top panel. Both systems exhibit multimodal distributions characteristic of mixed secondary structure, with φ peaks near −60° (α-helix) and −120° (β-strand), and ψ peaks near −30° α-helix and 120° β-strand. Holo displays a slight redistribution of ψ density toward lower angles, consistent with increased α-helical propensity. Circular variance (κ = 1 − mean resultant length) of the joint φ/ψ distribution used for quantifying conformational dispersion. Accordingly holo (0.52) and apo (0.53) show nearly identical angular spread. Box plots at lower panel summarize φ and ψ angle ranges. Median φ values are comparable, but the interquartile range of ψ is marginally narrower in holo (not shown), suggesting subtle restriction of ψ fluctuations.

The proportion of dihedral angles classified as steric outliers was marginally lower in the holo system (6.20%) compared to the apo system (6.97%). Analysis of the angular distributions showed that ligand binding was associated with a slight negative shift in the mean ψ angle (Holo: 44.2° ± 93.9°; Apo: 50.4° ± 92.0°), while the mean φ angle remained similar (Holo: −69.0° ± 72.3°; Apo: −67.8° ± 68.5°). The circular variance, a measure of angular spread, was comparable between the two states (Holo: 0.52; Apo: 0.53). Dihedral analysis revealed that SangfA binding induces some degree of local backbone adjustments without large-scale remodeling of the Ramachandran landscape (Fig 11).

### Ligand binding induces a statistically significant reorganization of secondary structure with a pronounced shift towards β-strand content

**Fig 12:**
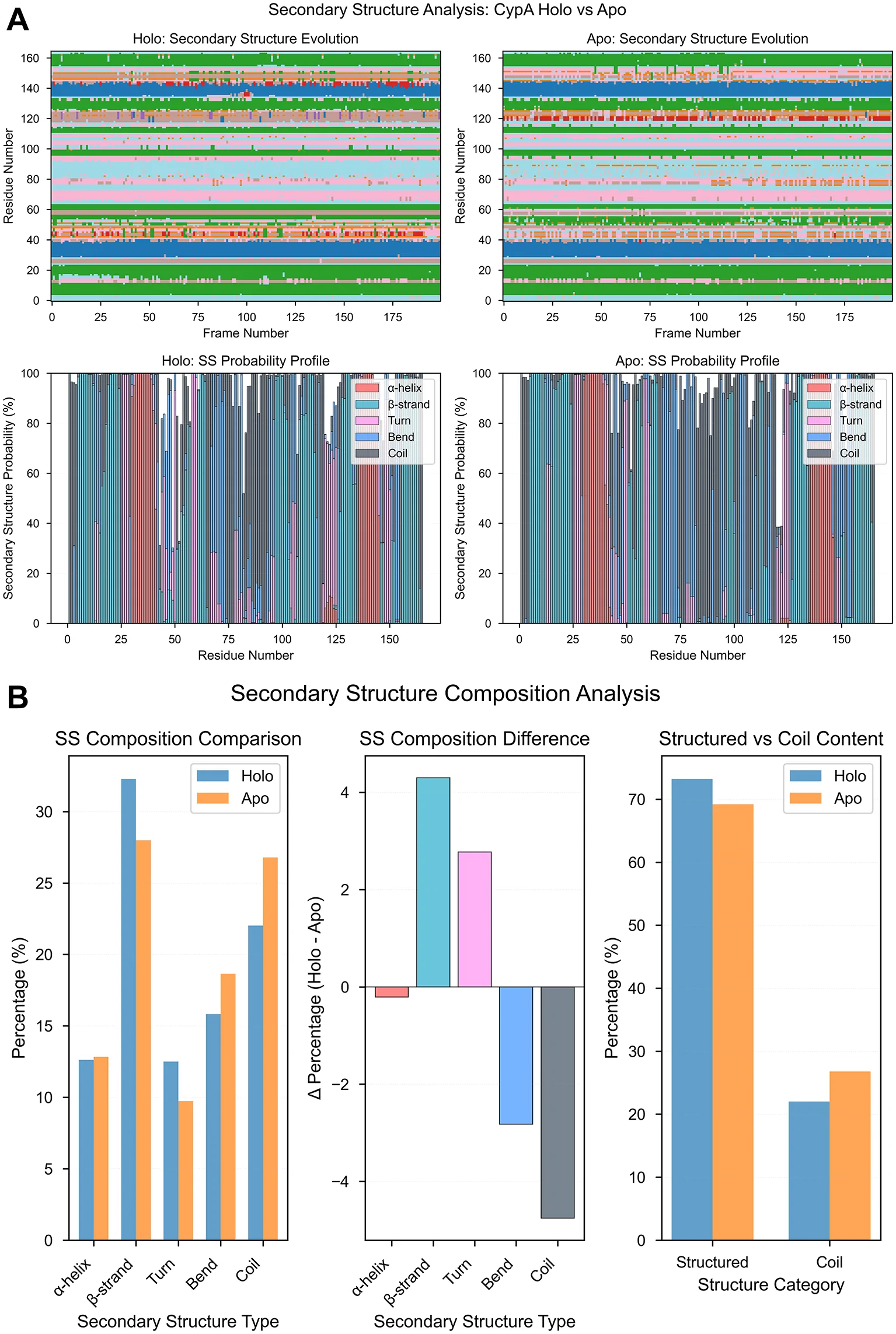
Secondary structure analysis of apo and holo CypA. (A) Per-residue secondary structure propensity and difference profile. Despite the global compositional shift, paired t-tests reveal no individually significant residues for α-helix (t = −0.06, p = 0.95) or β-strand (t = 0.94, p = 0.35) after multiple testing correction. Nevertheless, 40 of 165 residues (24.2%) undergo a change in the dominant secondary structure type upon binding. The most pronounced increases in helix propensity occur at residues 40 (Δ = 0.298), 122 (Δ = 0.088), and 123 (Δ = 0.084). Regional analysis highlights substantial secondary structure remodeling in the second half of the protein (residues 83–165), where β-strand content rises by 9.7% and coil content declines by 8.3%. These localized adjustments, particularly in the C-terminal half and active-site proximal regions, indicate that SangfA binding induces a subtle but widespread reorganization of CypA’s secondary structure landscape, favoring extended β-conformation at the expense of disordered loops while preserving the overall α/β fold. (B) Secondary structure composition. Bar chart showing the percentage of each secondary structure type (8,001 frames for apo, 8,002 frames for holo; 165 residues). Holo CypA exhibits a significant redistribution of secondary structure elements compared to apo. Specifically, holo shows decreased α-helix (H: 12.61% vs. 12.82%), increased β-sheet (E: 32.29% vs. 27.99%), reduced turn (T: 12.51% vs. 9.73%), and a marked decrease in random coil (~: 22.03% vs. 26.78%). Bend (S) and 3_10_-helix (G) fractions are also reduced in the holo state. The overall secondary structure stability index, average number of consecutive residues in the same state, is marginally lower in holo (6.30 vs. 7.15), suggesting slightly more dynamic secondary structure elements upon ligand binding.

Time-resolved secondary structure analysis (Fig 12) via DSSP revealed a significant compositional shift in CypA upon SangfA derivative binding (χ^2^ = 17776.30, p < 0.001). The holo state exhibited a marked increase in average β-strand content and a corresponding decrease in coil. The α-helix content remained relatively stable. This repartitioning was most pronounced in the C-terminal half of the protein, residues 83–165, which showed a net increase of 9.7% in β-strand content and an 8.3% decrease in coil (Table 4). Time-evolution analysis confirmed that the elevated strand content in the holo state was maintained consistently throughout the 100 ns simulation.

**Table 4:** Secondary structure composition by region for apo and holo CypA.

Region (residues)	Δ β-Strand (%)	Δ Coil (%)	Structural implication
Second Half (83–165)	9.7	−8.3	Major structuring of C-terminal domain
N-terminal (1–30)	−3.8	2.1	Mild destabilization
C-terminal (136–165)	0.9	−0.7	Minor compaction

### Specific residues in the binding pocket and catalytic loop undergo dramatic conformational transitions

**Fig 13:**
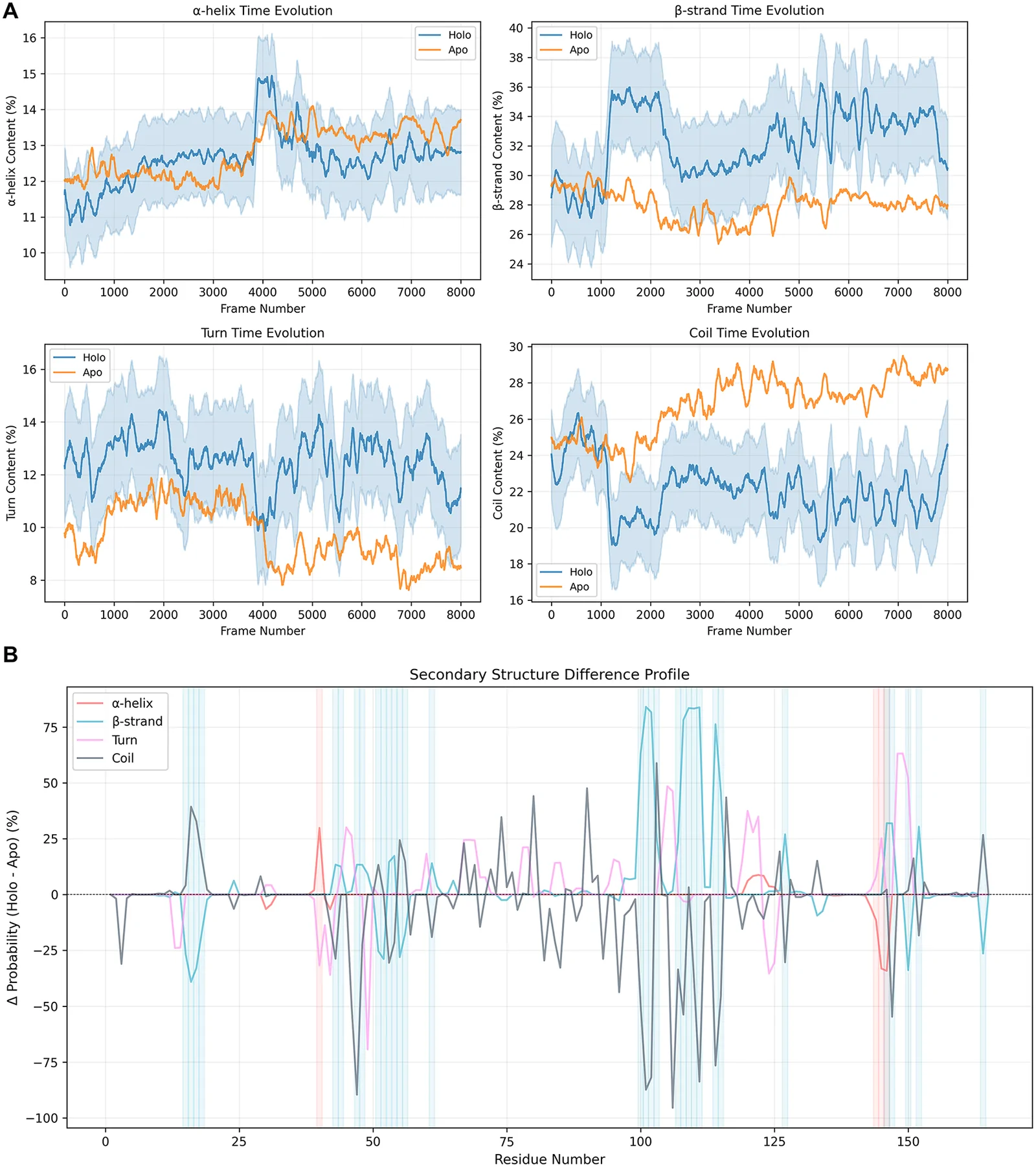
Time evolution of secondary structure content. Secondary structure composition of apo and holo as a function of simulation time. A) The time traces show that secondary structure contents, except turns, remain stable throughout the simulation after initial equilibration, with no systematic drift. Fluctuations in β-strand, coil, and turn contents are visibly larger in apo and holo systems. The holo system maintains persistently higher β-strand and lower coil fractions across nearly all frames, indicating that the observed conformational shift is not transient but represents a stable reorganisation of the CypA secondary structure landscape upon complex formation. Moreover, it is observed that most of the coil contents may have shifted toward turn rearrangements. B) The increase in β-strand and turn contents, and concomitant reduction in coil propensity upon ligand binding point to a local ordering of the CypA backbone, particularly in regions involved in substrate recognition, 80s loop, the 100–110 loop, and the C‑terminal β-strands. This secondary structure remodelling is consistent with a conformational selection mechanism, where SangfA preferentially binds a sub‑state of CypA featuring a more extended β-structure, thereby shifting the ensemble toward this conformation. The subtle but widespread nature of the changes underscores the capacity of small‑molecule ligands to modulate protein secondary structure without disrupting the global fold.

Per-residue analysis identified 40 residues that underwent a change in their dominant secondary structure type upon ligand binding. The most prominent transitions were localized to two key functional regions (Fig 13). One, the substrate-binding β10–β11 region, residues 100–111. Multiple residues exhibited a shift from coil or bend to well-defined β-strand or turn conformation in the holo state. Residues 100–102 and 108–111, which were mainly coil or bend in the apo state, adopted stable β-strand or turn conformations with near 100% probability in the holo state. This indicates a ligand-induced structuring and rigidification of a loop critical for substrate recognition. Second, the Catalytic and Adjacent Loops (Residues 145–146): Residue 145 transitioned from a helix in the apo state to a turn in the holo state (probability shift: 76.4% to 46.6%), while residue 146 shifted from a helix to a bend. This suggests a distortion or unwinding of a helical element near the active site upon ligand binding.

Analysis of conformational entropy and transition rates revealed a complex impact on local flexibility. While the overall structural content became more defined reduced coil, the effect on residue-wise dynamics was heterogeneous. Several residues in the binding vicinity like at residues 100, 101, and 102 (Fig 11B) showed a large decrease in conformational entropy and fewer secondary structure transitions in the holo state, indicating stabilization. Conversely, other regions, particularly in catalytic loops, residues 13–15, 41–43, displayed increased entropy and higher transition counts, suggesting ligand binding can introduce localized flexibility or dynamic disorder in specific loops, potentially facilitating functional motions.

### Binding free energy analysis using LIE method

The binding free energy of the protein-ligand complex was estimated using the LIE method. The average van der Waals (Lenard-Jones short-range) and electrostatic (Ciulombic short-range) interaction energies between CypA and SangfA were calculated to be −339.45 ± 33.38 kcal/mol and −48.92 ± 17.16 kcal/mol, respectively. Using empirical scaling factors α = 0.18 and β = 0.5, the LIE binding free energy was estimated to be ΔG_bind_ = −20.45 ± 3.07 kcal/mol).

### Ligand binding disperses conformational dynamics across more collective modes

**Fig 14:**
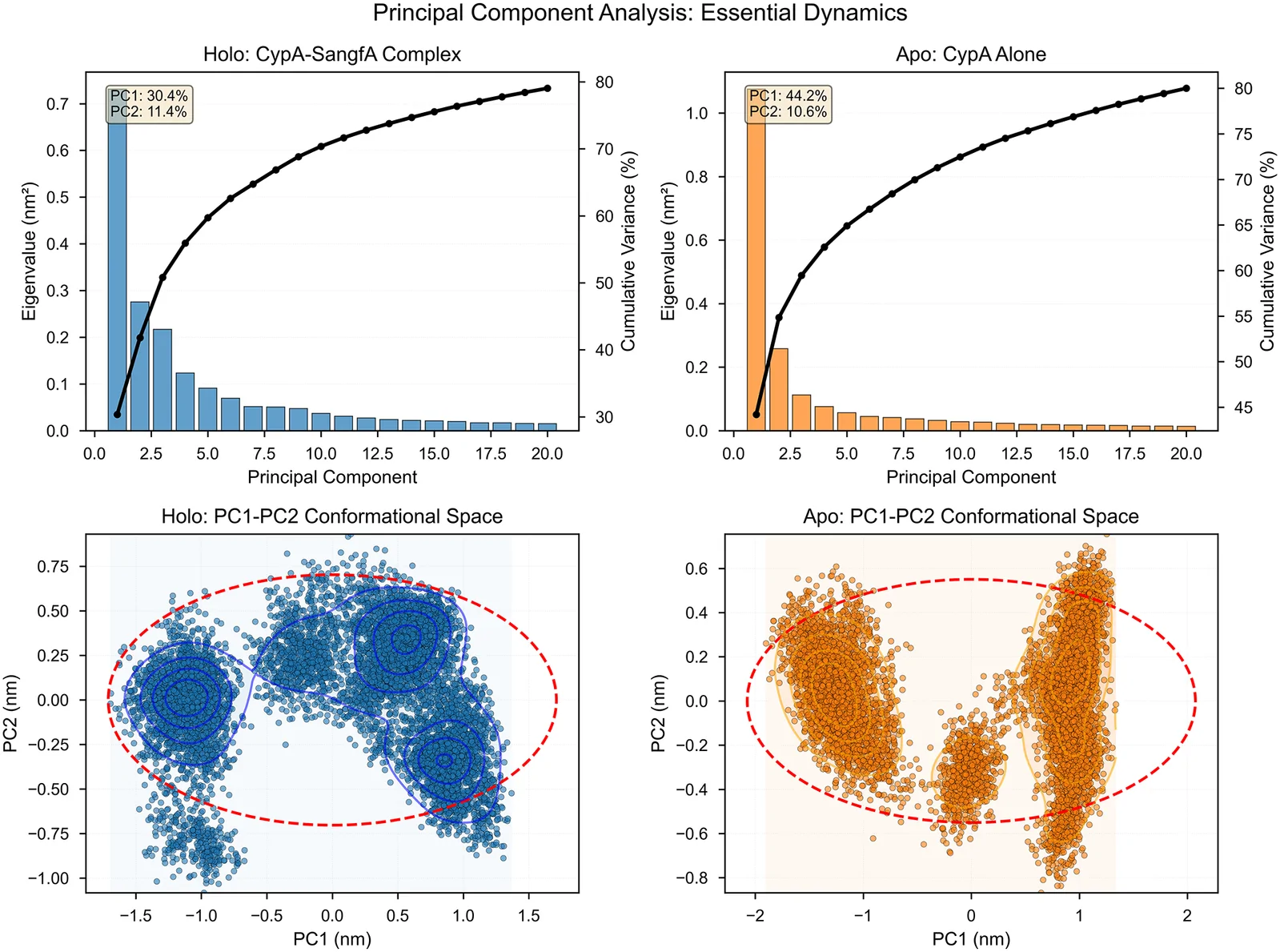
PCA analysis of CypA dynamics. (A) The covariance matrix of atomic coordinates was diagonalized to extract eigenvectors (PCs) and their associated eigenvalues, which represent the amplitude of motion along each collective degree of freedom. Two top plots show scree plots of eigenvalue spectrum. The first 20 eigenvalues are plotted for both systems. The initial eigenvalue decay is steeper for apo, indicating that its conformational space is dominated by fewer, large-amplitude collective motions. Specifically, PC1 captures the highest total variance in apo (eigenvalue = 1.076 nm^2^) but only 30.4% in holo (eigenvalue = 0.731 nm^2^). The second component accounts for 10.6% (apo) and 11.4% (holo). The cumulative variance explained by the first two PCs reaches 54.8% in apo versus 41.8% in holo; for the first ten PCs, the cumulative variance is 72.5% (apo) and 70.4% (holo). The more gradual eigenvalue decay in holo reflects a redistribution of motional amplitudes across a larger number of collective modes upon ligand binding. The lower two-dimensional projection plots are showing the conformational ensemble. Projection of the trajectories onto the first two PC1 vs. PC2 reveals distinct conformational distributions. The apo ensemble occupies a more extended region along PC1 (range: −1.2 to 1.8 nm) but is relatively confined along PC2. In contrast, the holo ensemble is more compact along PC1 but expands along PC2, resulting in a conformational volume of 0.301 nm^2^, compared to 0.285 nm^2^ for apo, a 5.6% increase. This indicates that SangfA binding does not simply rigidify CypA; instead, it remodels the accessible conformational space, permitting broader exploration along previously restricted directions while suppressing the dominant large‑amplitude mode.

PCA of Cα atomic fluctuations reveals that the SangfA significantly alters the collective motion profile of CypA. In the apo state, the first principal component (PC1) dominates, capturing 44.2% of the total positional variance (eigenvalue: 1.076 nm^2^). In contrast, in the holo complex, the variance is distributed more evenly across multiple modes, with PC1 explaining only 30.4% of the variance (eigenvalue: 0.731 nm^2^) (Fig 14). Consequently, more PCs are required to describe an equivalent fraction of the total motion in the holo state; the first two PCs account for 54.8% of variance in apo but only 41.8% in holo. The participation ratio for PC1 was 8.3 in the holo system, indicating that the dominant collective motion is highly localized, likely involving only a few residues or secondary structure elements. This contrasts with typical global domain motions in CypA and suggests that ligand binding focuses essential dynamics into functionally relevant, spatially restricted regions. The PCA demonstrates that formation of the CypA-SangfA complex leads to a suppression of the largest‑scale concerted motions, a redistribution of conformational diversity across more modes, and an overall expansion of the explored conformational space, consistent with a shift from a more ordered, “tunneled” apo landscape to a broader, more heterogeneous holo ensemble. This dynamic remodeling may facilitate substrate recognition and turnover in the native catalytic cycle.

### The holo state explores a broader but differently shaped conformational landscape

**Fig 15:**
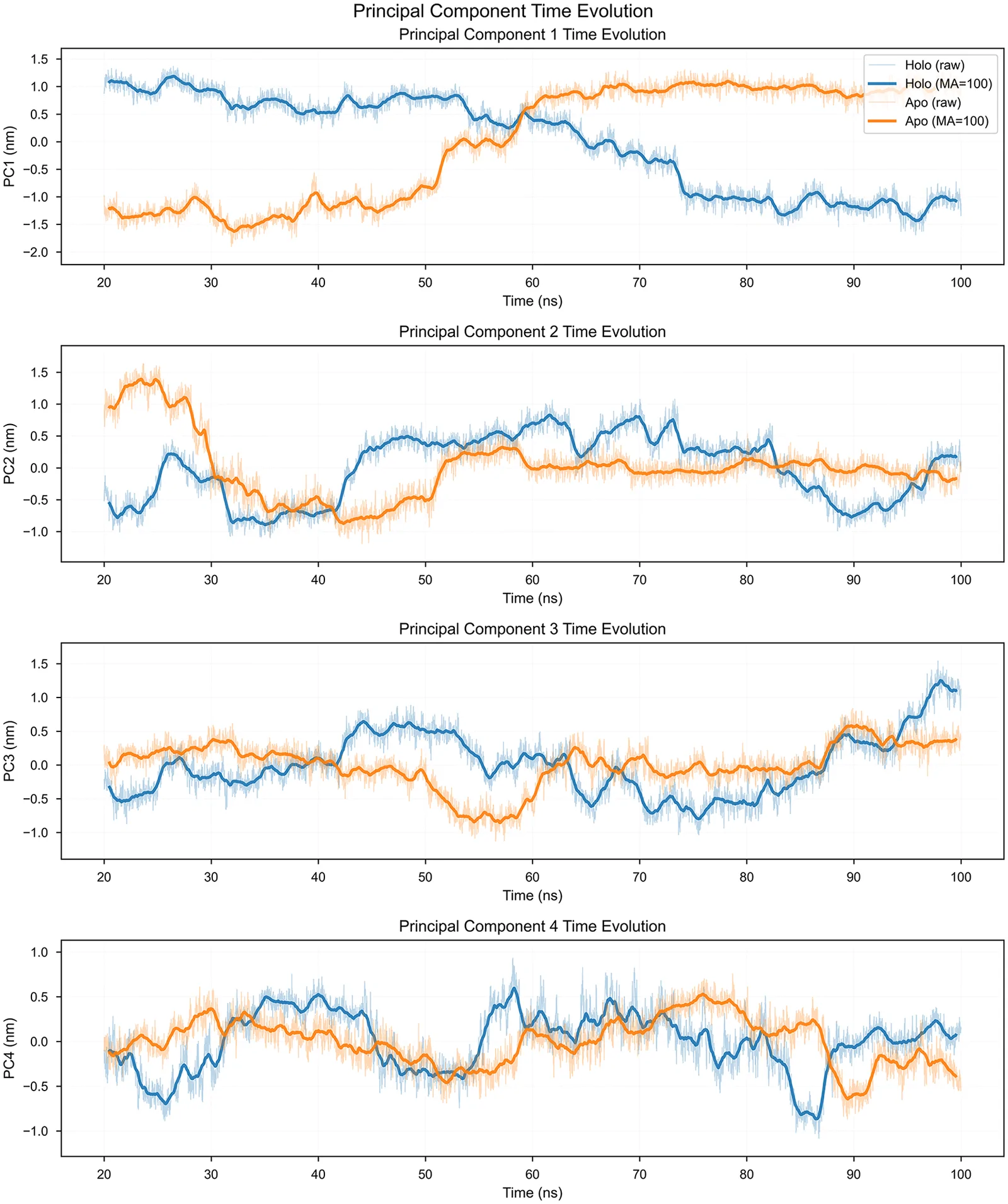
PCs’ time evolution. Time series of the projections of the MD trajectories onto the first four PC1-PC4 for apo and holo CypA. Raw data are shown as faint, semi‑transparent lines; a 100‑frame moving average (MA = 100, corresponding to 1.25 ns) is overlaid as solid curves to highlight low‑frequency trends. Top plot PC1 projection: The amplitude of PC1 fluctuations is markedly larger in apo (range: < −1.0 to 1.8 nm) than in holo (range: −0.8 to 1.2 nm). The moving average reveals persistent excursions in apo that are absent in the complex, indicating that ligand binding dampens the principal collective mode, assigned to a breathing motion of the β-barrel by 40%. PC2 projection: While the raw fluctuation range is similar between the two systems, the apo trajectory exhibits more frequent high‑amplitude spikes and a broader distribution of PC2 values. The holo projection appears more stationary and less responsive to fast fluctuations, suggesting stabilization of the conformational substate ensemble along this coordinate. PC3 and PC4 projections: These higher‑order modes, which collectively account for 13% of the total variance in both systems, show comparable fluctuation amplitudes. However, the apo traces display occasional abrupt transitions that are not mirrored in the holo trajectory.

Projection of the trajectories onto the first two eigenvectors shows distinct conformational sampling (Fig 15). While the magnitude of motion along the primary mode (PC1) is reduced in the holo state (0.855 nm vs. 1.037 nm), the motion along PC2 is increased (0.352 nm vs. 0.275 nm). The overall area of the PC1–PC2 subspace, a measure of conformational space sampled, is 1.1 times larger for the holo complex (Holo: 0.301; Apo: 0.285). This indicates that ligand binding does not simply constrain global motion but rather redirects it, trading amplitude in the dominant mode for exploration along orthogonal, secondary coordinates. The time evolution of the PCs demonstrates that SangfA binding selectively attenuates the amplitude and frequency of large‑scale concerted motions, particularly those represented by PC1 and PC2, while preserving or even enhancing the exploration of orthogonal conformational subspaces. This dynamic fingerprint is characteristic of a conformational selection mechanism, wherein the ligand stabilizes a pre‑existing sub‑state and reshapes the free‑energy landscape to favor functionally relevant fluctuations. The collectivity of the dominant motions, quantified by the participation ratio, is lower in the holo state (8.3). A lower value indicates that the motions described by the first few eigenvectors involve a smaller subset of the protein’s residues. This suggests that the principal dynamics in the ligand-bound state are more localized, potentially focusing on regions around the binding site or specific flexible loops, whereas the apo state’s dominant motions are more globally collective.

## Discussion

Prior research has established the importance of understanding the role of CypA and its inhibition in infectious diseases [50–52]. Accordingly, the protective role of CypA in influenza A virus infections in transgenic mice has been demonstrated, highlighting the protein’s implication in immune response modulation [53]. Furthermore, computational methods have also been used to study the full electron structure of the CypA-CsA complex, providing insights into the molecular interactions at the electron level, which is complementary to our MD simulation approach focusing on dynamic structural changes [54]. In this study, an all-atom MD simulation was performed to investigate the interactions between CypA and SangfA. The results demonstrated that SangfA has a high stable affinity for CypA, leading to significant structural and dynamic changes in the protein.

In the present study, it was observed that SangfA binding exerts nuanced effects on CypA structure and dynamics. Heal et al. (2015) highlighted the importance of hydrogen bonds in the stability of the CypA-CsA complex [55]. The high-resolution CypA-SangfA crystal structure [56] shows the macrocyclic portion of SangfA deeply buried in the CsA-like pocket with six hydrogen bonds to the protein. We found that in water solution these interactions are highly dynamic. At any instant only one or two strong H‑bonds persist, while waters constantly mediate others. This is unsurprising given the solvation of the binding site, but it implies that the static network of six H-bonds becomes a flickering ensemble of transient contacts during MD. Nonetheless, the ligand remains tightly bond, mirroring the crystallographic deep embedment [56]. Moreover, while the crystallographic model reported by Kallen et al. [56] depicts a tightly hydrogen-bonded ligand deeply embedded in the pocket, our trajectories reveal that these interactions are highly dynamic, with transient, solvent-mediated contacts maintaining stable binding. Consistent with Eisenmesser et al.’s [57] identification of the L98-S99 region as a catalytically mobile loop, SangfA induces β-strand formation and local rigidification in this segment, providing a mechanistic explanation for enzymatic inhibition. PC and clustering analyses further support a conformational selection model, as the holo ensemble explores a broader yet redistributed landscape with increased configurational entropy. Together, these results show that SangfA inhibits CypA not by rigid locking but by selectively reshaping its dynamic ensemble.

Mechanistically, SangfA binding does not simply lock down CypA rigidly. Instead, the RMSD (Fig 1) and RMSF (Fig 2) results indicate a modest increase in global deviation but a narrower distribution, meaning the holo protein samples a somewhat higher-energy conformation more consistently. The RMSF analysis showed increased fluctuations in specific residues upon SangfA binding in amino acid residues Met1, Asp13-Leu17, Arg69-Thr73, and Glu81-Asp85, which may be crucial for the optimal positioning of the ligand within the binding pocket [50]. The reduced fluctuations also observed in the Thr41-Lys49, Ala100-Gly111 regions of the holo system. The ligand remains in close proximity throughout the simulation (Fig 6), with an average minimum distance of 0.199 nm and 100% contact occupancy, confirming a tightly bound complex. These results indicate that ligand binding induces conformational changes necessary for CypA’s function [58]. Equivalently, PCA analysis showed that binding spreads out fluctuations over more modes, producing a broader conformational ensemble. In other words, ligand-bound CypA appears to follow a conformational selection model, and SangfA shifts the equilibrium to a different set of pre-existing protein states rather than inducing an entirely new conformation. This view is supported by previous study, which found that substrate-bound active-site dynamics are largely inherent to the free enzyme [59]. Our findings show that CypA in the holo state visits more distinct clusters, 75 vs. 66, and a wider PC1-PC2 area than apo, suggesting that SangfA binding accesses alternate substates of the protein. Also, increased SASA value upon SangfA binding (Fig 3) highlights that SangfA makes CypA more accessible to solvate in favor of solvation. Residues including Phe46, Thr73, Lys76, Ile89, Lys91, and Thr93 increased solvability in the complex system. Binding a ligand frequently changes the protein’s conformation, exposing hidden hydrophobic or hydrophilic residues to the surrounding solvent. This is probably because the protein changes to a more open shape to fit the ligand, increasing the SASA of these residues [53,60]. Moreover, residues Ile89, Lys91, and Thr93 are critical for maintaining the protein’s overall conformation and stability [61,62], and possibly the structural rearrangements induced by SangfA increase the solvent accessibility of these residues. The increased solvent exposure could facilitate the binding and release of substrates or inhibitors, contributing to CypA’s overall catalytic efficiency. On the other hand, Glu34, Gly42, and Phe60 displayed reduced solvent exposure upon SangfA binding, suggesting that these residues are more enclosed within the protein-ligand complex, possibly enhancing the complex’s stability.

Importantly, the ligand exerts localized rigidity at the expense of flexibility elsewhere. The most dramatic effect was at the C-terminal β2–β3 loop, residues 100–102 and 108–111 became β-stranded most of the times when SangfA was bound. This loop flanks Leu98-Ser99, a region previously identified as a dynamic site during catalysis [57,63]. Thus, SangfA appears to stabilize the conformation of this loop, possibly locking one end of the active-site tunnel. At the same time, certain distal loops became more mobile. Such reorganization suggests an entropic trade-off. Accordingly, by clamping the substrate-access loop, the protein compensates by loosening other segments. This dynamic redistribution may be mechanistically relevant. For example, the newly formed β-strand around residue 101 (Fig 8A) could restrict entry to the catalytic center, complementing Arg55’s anchoring of the ligand. Yet because Arg55 itself did not contact SangfA in our simulation, the proline-recognition site may remain partially accessible. In fact, Arg55 is known to anchor substrate prolines [64], so its absence in the ligand network hints that SangfA’s effector domain leaves the catalytic machinery only partially occluded. This could explain previous suggestion that SangfA’s effector differs fundamentally from CsA’s [56], perhaps preserving some residual isomerase activity.

From a drug-design standpoint, the interplay between structure and dynamics is significant [65–67]. SangfA is already recognized as an exceptionally potent cyclophilin inhibitor [68,69]. SangfA exhibited high affinity towards CypA, showing an IC50 value of 6.9 ± 0.9 nM [70]. The main interaction is mainly controlled by the macrocyclic region of the SangfA molecule, which binds deeply into the hydrophobic pocket of CypA, much like how CsA binds. Similarly, the binding free energy calculations using the LIE method provided an estimated ΔG_bind_ of −20.45 ± 3.07 kcal/mol, indicating a strong binding affinity of SangfA. The detailed dynamics, however, reveal how flexibility might be harnessed in future analogs. The fact that SangfA increases CypA’s conformational entropy and alters H‑bond persistence suggests that ligand design should consider not just static contacts but also how the ligand drives protein fluctuations. For instance, the strong role of Lys105 and Phe102 in binding, from the contact analysis (Fig 5) and charged residue statistics (Table 1), may guide modifications to the macrocycle or effector to target these underappreciated subsites. Moreover, it has been shown that residues adjusted in the binding site, including Arg55, Phe60, and Trp121, significantly contributed to the PCs. These residues are crucial for the binding and stabilization of SangfA. Arg55, in particular, was identified as a key player in the hydrogen bonding network that stabilizes the CypA-SangfA complex [54,58]. The motion of these residues is essential for accommodating the ligand and facilitating effective inhibition. Also, the induced β-strand in the β2-β3 loop suggests that reinforcing this stabilization or preventing it could modulate inhibitor potency or specificity. To complement the quantitative analysis, a Supplementary S1 Video is provided, which synchronizely animate the full 100 ns MD trajectories of apo and holo CypA systems. Future NMR and X-ray crystallographic experiments can be conducted to validate the predicted conformational switch in the β2-β3 loop on the CypA-SangfA complex would be invaluable. Especially, the findings on the transition of residues Ile97-Asn10 from coil in apo system to a β-sheet in holo system warrants further experimental validation. Additionally, structure-guided design, pharmacophore modeling, virtual screening, and synthesis of SangfA analogs that contact with Lys105 or that reinforce the coil-to-sheet transition in the 100–111 segment could yield inhibitors with tailored dynamic profiles and improved pharmacological properties.

Biologically, cyclophilin A mediates diverse processes, from viral infection to cancer, via both its isomerase activity and protein–protein interactions. Our findings imply that Sanglifehrin’s immunosuppressive effects may involve both tight binding and subtle modulation of CypA dynamics. By tuning the active-site loop conformation, SangfA might affect how CypA interacts with partners such as viral capsid proteins. Indeed, the loop around Phe102/Trp121 contacts the HIV capsid [59,71]. If SangfA locks that loop differently, it could alter capsid recognition. On the translational side, the insight that ligand binding *increases* overall protein flexibility might encourage drug developers to screen not only for static affinity but also for compounds that achieve the desired dynamic profiles. High-affinity CypA ligands that overly rigidify the protein could have off-target effects, whereas those that maintain an optimal degree of plasticity might yield better safety.

The results of the presented study demonstrated that SangfA imposes a unique dynamic fingerprint on CypA. The ligand forms a tightly bound yet dynamic hydrogen-bond network and anchors to Arg55, but without fully inhibiting the conformational breathing of the enzyme. The ligand binding stabilizes key loop regions near L98-S99 by inducing β-structure, while simultaneously enhancing motions in other loops, a balancing act that increases the protein’s configurational entropy. This nuanced modulation likely underlies SangfA’s potent inhibition and suggests new avenues for designing cyclophilin-targeted drugs.

One major constraint of this research is the sole dependence on MD simulation to anticipate the interaction between CypA and SangfA. Although MD simulation offers an in-depth understanding of the structural and dynamic characteristics of the protein-ligand complex, it needs to consider the biological complexities and cellular environments in which these interactions occur. Also, due to the employed forcefield, gromos43a1.ff, we were not able to perform Mechanics-Poisson Boltzman Surface Area (MM-PBSA) analysis. This will be addressed in a future study.

## Conclusion

In this study, we employed extensive all-atom MD simulations to dissect the structural and dynamic consequences of SangfA binding to CypA. Our results reveal that SangfA does not simply lock CypA into a single rigid conformation. Instead, it imposes a nuanced dynamic fingerprint characterized by localized rigidification of the active‑site where residues 100–102 and 108–111 undergo a coil‑to‑β-strand transition, coupled with enhanced mobility in distal loops and an overall expansion of the accessible conformational ensemble. The ligand engages CypA through a flickering hydrogen‑bond network, anchored persistently by Arg55 and supplemented by hydrophobic contacts with Phe60 and Lys105, while the canonical proline‑recognition residue Arg55 remains uninvolved. PCA demonstrated that the ligand binding redistributes collective motions from a single dominant mode to a broader spectrum of fluctuations, increasing configurational entropy and the number of distinct conformational clusters. These observations support a conformational selection mechanism that is characterized by heightened β-sheet content in the substrate‑binding loop and a more heterogeneous motional landscape. Our findings carry direct implications for the design of next‑generation cyclophilin inhibitors. The induced β-strand around residues 100–102 represent unexploited structural motifs that could be targeted to enhance selectivity or modulate the dynamic response. Also, the increased CypA’s conformational upun SangfA bonding challenges the traditional rigid‑body view of inhibition and suggests that functional outcomes may be governed by the reshaping of the protein’s energy landscape rather than by simple steric blockade. Future experiments can be conduct to validate the predicted conformational switch in the β2-β3 loop on the CypA-SangfA complex would be invaluable. Additionally, structure‑guided design, pharmacophore modeling, virtual screening, and synthesis of SangfA analogs that contact with Lys105 or that reinforce the coil‑to‑sheet transition in the 100–111 segment could yield inhibitors with tailored dynamic profiles and improved pharmacological properties.

## Supporting information

S1 Data The raw data of gromacs analysis pipeline used for this study. (ZIP)

S1 Video Side-by-side synchronized comparison of apo (left panel) and holo (right panel) trajectories, allowing direct visual comparison of conformational differences. Full 100 ns MD trajectory of apo and holo CypA systems rendered with VMD and encoded at 25 fps on 1002 frames.
(MP4)
