# Supplementary material for: All-atom molecular dynamics simulation of Cyclophilin A in complex with Sanglifehrin A
Source: PLoS One. 2026 Sep 22;21(9):e0358847. doi: 10.1371/journal.pone.0358847 (PMC13596834; doi:10.1371/journal.pone.0358847)
Supplement: Supplementary file 1 [file pone.0358847.s004.docx]

**June 5, 2026**

**Dear Editorial of Plos One,**

Thank you for your careful review of our manuscript and for the opportunity to revise our supporting information files in accordance with PLOS ONE’s data accessibility guidelines.

We acknowledge the concern raised regarding the supporting information file run_basic.bat. As you correctly note, a script file does not meet the requirement for data to be provided in a standard, accessible format from which data can be efficiently extracted.

To fully comply with the journal’s policy, we have made the following changes:

1. Removed the run_basic.bat file from the Supporting Information.
2. Provided the actual numerical data generated by the simulations in a standard, reusable format.

We have now converted all GROMACS output files (.xvg) into comma‑separated values (CSV). All CSV files are organized in a ZIP archive named S1_Data.zip, with a clear directory structure separating the Holo and Apo systems. The archive includes all analysis outputs (RMSD, RMSF, SASA, hydrogen bonds, contacts, PCA, DSSP, contact maps, clustering, salt bridges, and protein–ligand interactions), as summarized in the updated Supporting Information caption.

3. Ensured the data are accessible and machine‑readable.
4. Updated the manuscript accordingly.

The batch script is no longer listed or cited.

We believe that these revisions fully address the journal’s requirement for data in an accessible, reusable format. The ZIP archive contains only the processed numerical data, with no scripts or executables. The file size is well under the 10 MB recommended limit.

Thank you for handling our submission.

Sincerely,

Alireza Mohebbi
