# Supplementary material for: All-atom molecular dynamics simulation of Cyclophilin A in complex with Sanglifehrin A
Source: PLoS One. 2026 Sep 22;21(9):e0358847. doi: 10.1371/journal.pone.0358847 (PMC13596834; doi:10.1371/journal.pone.0358847)
Supplement: Supplementary file 2 [file pone.0358847.s005.docx]

**September 4, 2026**

**Manuscript Title:** All-Atom Molecular Dynamic Simulation of Cyclophilin A in Complex with Sanglifehrin A

**Dear Editorial of PLOS ONE,**

Thank you and the reviewers for the careful review of our manuscript and for the opportunity to revise our manuscript. Below, we have addressed the reviewers concerns and recommendations thoroughly point-by-point.

**Reviewer 1**

**Comment:** "Dear authors, Thank you very much for addressing all my questions. The authors should recommend NMR or X-ray crystallographic studies to verify their findings. The tables and figures should be cited in the discussion in my opinion."

**Authors' Response:** We thank Reviewer for their positive assessment and constructive suggestions. We have incorporated both recommendations into the revised manuscript:

1. **Recommendation of experimental validation:** We have added a paragraph in the Discussion section explicitly recommending NMR and X-ray crystallographic studies to experimentally validate our computational predictions. We now state:

*"* *Future NMR and X-ray crystallographic experiments can be conducted to validate the predicted conformational switch in the β2-β3 loop on the CypA-SangfA complex would be invaluable. Especially, the findings on the transition of residues Ile97-Asn10 from coil in apo system to a β-sheet in holo system warrants further experimental validation. Additionally, structure-guided design, pharmacophore modeling, virtual screening, and synthesis of SangfA analogs that contact with Lys105 or that reinforce the coil-to-sheet transition in the 100-111 segment could yield inhibitors with tailored dynamic profiles and improved pharmacological properties."*

2. **Citation of tables and figures in the Discussion:** We have carefully revised the Discussion section to ensure that tables or figures are cited where it was possible.

**Reviewer 2**

**Comment:** "Yes. The manuscript is generally intelligible and written in understandable English. However, minor typographical and grammatical errors remain and should be corrected during final editorial polishing."

**Authors' Response:** We thank dear Reviewer for careful reading. We have performed a thorough proofreading of the entire manuscript and corrected all identified typographical and grammatical errors. Specific corrections include:

- Line 15: "basid MD analyzes" → "basic MD analyses"
- Line 45: "escalated an opportunistic viral and bacterial co-infection" → "escalated an opportunistic viral and bacterial co-infection" (corrected)
- Line 66: Removed redundant re-definition of "molecular dynamics (MD)"
- Line 97: "at 300°K" → "at 300 K"
- Line 111: "implemented to compared" → "implemented to compare"
- Line 484: "Teses indicate that ligand binding..." → "These results indicate that ligand binding..."

We have also conducted a comprehensive language polish throughout the manuscript to improve clarity and readability.

**Reviewer 4**

**Comment:** "As on revision, manuscript is quite polished and can be considered for publication upon minor checks and reading."

**Authors' Response:** We thank Reviewer 4 for their positive assessment and recommendation for publication. We have performed a final careful reading and formatting check of the entire manuscript to ensure all minor issues are resolved.

**Reviewer 5**

**Comment 1: "Please provide a figure with the most representative structures found during the simulations, pointing out relevant domains and residues."**

**Authors' Response:** We thank Reviewer for this valuable suggestion. We have added two new figure (Figure 8 and Figure 9) to the revised manuscript that presents the most representative structures from the simulations, with relevant domains and residues clearly labeled.

We have also updated the figure legend to provide full details on the structural features shown and their significance.

**Comment 2: "Please provide an animation or some other means to visualize the motions associated with the main principal components. Examples can be found in Martínez-Archundia, M., Correa-Basurto, J., Montaño, S., & Rosas-Trigueros, J. L. (2019). Studying the collective motions of the adenosine A2A receptor as a result of ligand binding using principal component analysis. Journal of Biomolecular Structure and Dynamics."**

**Authors' Response:** We sincerely thank Reviewer #5 for this valuable suggestion. We agree that visualizing the protein motions provides crucial insight into the conformational dynamics described in our study. In response to this comment, we have generated a supplementary video (Supplementary Videos S1) from our MD trajectories that directly address this request:

**Direct visualization of PC1 and PC2 motions:** The dominant collective motions identified through PCA correspond to the large-amplitude conformational rearrangements that are clearly visible in these animations.

However, the β2-β3 loop, which undergoes a coil-to-β-strand transition upon ligand binding is now presented in Figure 8 and Figure 9, allowing the reader to correlate the secondary structure changes with the observed dynamics.

**Comment 3: "In line 95: The system was then neutralized by Cl⁻ or Na⁺ ions. Please report the nature and number of the ions used."**

**Authors' Response:** We apologize for this omission. The revised manuscript now explicitly states the nature and number of ions used for neutralization. In the Methods section, we now write:

*"Both apo and holo systems were then neutralized by adding 2 Cl^-^ ions …"*

**Comment 4: "In line 128, Essential Dynamics and Conformational Sampling. Equations should be numbered and discussed in the text."**

**Authors' Response:** We have revised the Essential Dynamics section to ensure all equations are properly numbered and discussed in the text. Further explanation of each equation is not included.

**Comment 5: "They write 'The first few principal components (PCs) with the largest eigenvalues (>80%)'. Please state how many PCs were used and better explain the 80% criterion."**

**Authors' Response:** We thank the reviewer for catching this ambiguity. In the revised manuscript, we now write:

*"The first four principal components (PCs) with the largest eigenvalues were computed, capturing 83.42% …”.*

**Comment 6: "In line 150 Calculation of Linear Interaction Energy (LIE). Same problem with the equations. Their reference [51] does a good job writing these equations, they should follow this reference more closely."**

**Authors' Response:** We have thoroughly revised the LIE section to follow the formalism of reference [51] (Rifai et al., 2020) more closely. The revised Methods section now presents the equations in a clearer form:

We have added explanatory text following each equation to clarify the physical meaning of each term.

**Comment 7: "I'm confused. In line 510 they write 'complementing Asp55's anchoring of the ligand. Yet because Arg55...'. So, is residue 55 Asp or Arg?"**

**Authors' Response:** We sincerely apologize for this typographical error and the confusion it caused. Residue 55 in Cyclophilin A is Arginine (Arg55), as correctly identified in our contact analysis reports and structural data (Table S1, Table S3, and residue-level contact analysis). The mention of "Asp55" was an unintentional typo; it should have read "Arg55" throughout that sentence. We have corrected the manuscript accordingly.

We appreciate the reviewer's diligence in catching this error.

**Comment 8: "Other minor issues follow: 15 Beside basid MD analyzes... basic MD analyses?"**

**Authors' Response:** We apologize for these typographical errors. In the revised manuscript

**Comment 9: "45 escalated the opportunistic... escalated an opportunistic"**

**Authors' Response:** Corrected. The revised manuscript (page 3, line 45) now reads:

*"It has been reported that CypA* ***escalated an opportunistic*** *viral and bacterial co-infection [16]."*

**Comment 10: "In line 13 they define molecular dynamics (MD), and later in line 66 they re-define molecular dynamic (MD)... It is my perception that the most accepted variation is Molecular Dynamics Simulations, either as MD or MDS."**

**Authors' Response:** We thank the reviewer for this clarification. We have revised the manuscript. The redundant re-definition was removed at line 66, which now simply uses "MD simulation" without re-defining the abbreviation.

We have also used the full term "Molecular Dynamics Simulations" consistently throughout the manuscript where appropriate.

**Comment 11: "97 at 300°K... at 300 K"**

**Authors' Response:** Corrected. The revised manuscript now states:

**Comment 12: "111 implemented to compared... implemented to compare"**

**Authors' Response:** Corrected. The revised manuscript now reads:

*"...implemented to* ***compare*** *the holo form …"*

**Comment 13: "484 Teses indicate that ligand binding... Consider rewriting."**

**Authors' Response:** We have rewritten this sentence for clarity.

**Additional Editor Comments**

**Editor Comment:** "Thanks for submitting the revised manuscript and the revised manuscript improved notably. However, it needs to address some minor comments of the reviewers, especially reviewer #5."

**Authors' Response:** We thank the Academic Editor for the positive assessment and for the opportunity to further improve our manuscript. We have addressed all comments raised by dear Reviewers in detail, as described above.

**Journal Requirements**

**"Please review your reference list to ensure that it is complete and correct. If you have cited papers that have been retracted, please include the rationale for doing so in the manuscript text, or remove these references and replace them with relevant current references."**

**Authors' Response:** We have carefully reviewed our complete reference list. All references are current, correct, and properly formatted. We have confirmed that none of the cited papers have been retracted.

We hope that these revisions adequately address all comments raised by the reviewers and the Academic Editor. We believe the manuscript is now significantly improved and suitable for publication in PLOS ONE. We thank all reviewers for their constructive feedback, which has helped us strengthen the clarity, rigor, and completeness of our work.

**Corresponding Author:** Alireza Mohebbi, PhD

**Email:** alirezaa2s@gmail.com
