# Supplementary material for: All-atom molecular dynamics simulation of Cyclophilin A in complex with Sanglifehrin A
Source: PLoS One. 2026 Sep 22;21(9):e0358847. doi: 10.1371/journal.pone.0358847 (PMC13596834; doi:10.1371/journal.pone.0358847)
Supplement: Peer review history

# Peer review: Decision Letter 0

**DOI:** 10.1371/journal.pone.0358847.r001

**Authors:** Farhadul Islam

19 Nov 2025

Dear Dr. Mohebbi,

Thank you for submitting your manuscript to PLOS ONE. After careful consideration, we feel that it has merit but does not fully meet PLOS ONE’s publication criteria as it currently stands. Therefore, we invite you to submit a revised version of the manuscript that addresses the points raised during the review process.

Please submit your revised manuscript by Jan 03 2026 11:59PM. If you will need more time than this to complete your revisions, please reply to this message or contact the journal office at plosone@plos.org. When you're ready to submit your revision, log on to https://www.editorialmanager.com/pone/ and select the 'Submissions Needing Revision' folder to locate your manuscript file.

- A rebuttal letter that responds to each point raised by the academic editor and reviewer(s). You should upload this letter as a separate file labeled 'Response to Reviewers'.
- A marked-up copy of your manuscript that highlights changes made to the original version. You should upload this as a separate file labeled 'Revised Manuscript with Track Changes'.
- An unmarked version of your revised paper without tracked changes. You should upload this as a separate file labeled 'Manuscript'.

If you would like to make changes to your financial disclosure, please include your updated statement in your cover letter. Guidelines for resubmitting your figure files are available below the reviewer comments at the end of this letter.

If applicable, we recommend that you deposit your laboratory protocols in protocols.io to enhance the reproducibility of your results. Protocols.io assigns your protocol its own identifier (DOI) so that it can be cited independently in the future. For instructions see: https://journals.plos.org/plosone/s/submission-guidelines#loc-laboratory-protocols. Additionally, PLOS ONE offers an option for publishing peer-reviewed Lab Protocol articles, which describe protocols hosted on protocols.io. Read more information on sharing protocols at https://plos.org/protocols?utm_medium=editorial-email&utm_source=authorletters&utm_campaign=protocols.

We look forward to receiving your revised manuscript.

Kind regards,

Farhadul Islam, PhD

Academic Editor

PLOS ONE

Journal Requirements:

When submitting your revision, we need you to address these additional requirements.

1. Please ensure that your manuscript meets PLOS ONE's style requirements, including those for file naming. The PLOS ONE style templates can be found at

https://journals.plos.org/plosone/s/file?id=wjVg/PLOSOne_formatting_sample_main_body.pdf and https://journals.plos.org/plosone/s/file?id=ba62/PLOSOne_formatting_sample_title_authors_affiliations.pdf

2. Please note that PLOS One has specific guidelines on code sharing for submissions in which author-generated code underpins the findings in the manuscript. In these cases, we expect all author-generated code to be made available without restrictions upon publication of the work. Please review our guidelines at https://journals.plos.org/plosone/s/materials-and-software-sharing#loc-sharing-code and ensure that your code is shared in a way that follows best practice and facilitates reproducibility and reuse.

3. We note that your Data Availability Statement is currently as follows: [All relevant data are within the manuscript and its Supporting Information files.]

Please confirm at this time whether or not your submission contains all raw data required to replicate the results of your study. Authors must share the “minimal data set” for their submission. PLOS defines the minimal data set to consist of the data required to replicate all study findings reported in the article, as well as related metadata and methods (https://journals.plos.org/plosone/s/data-availability#loc-minimal-data-set-definition).

For example, authors should submit the following data:

- The values behind the means, standard deviations and other measures reported;

- The values used to build graphs;

- The points extracted from images for analysis.

Authors do not need to submit their entire data set if only a portion of the data was used in the reported study.

If your submission does not contain these data, please either upload them as Supporting Information files or deposit them to a stable, public repository and provide us with the relevant URLs, DOIs, or accession numbers. For a list of recommended repositories, please see https://journals.plos.org/plosone/s/recommended-repositories.

If there are ethical or legal restrictions on sharing a de-identified data set, please explain them in detail (e.g., data contain potentially sensitive information, data are owned by a third-party organization, etc.) and who has imposed them (e.g., an ethics committee). Please also provide contact information for a data access committee, ethics committee, or other institutional body to which data requests may be sent. If data are owned by a third party, please indicate how others may request data access.

4. Thank you for stating the following in the Competing Interests section:

[The authors have declared that no competing interests exist.].

We note that one or more of the authors are employed/affiliated by a commercial company: Vista Aria Rena Gene Inc.

1. Please provide an amended Funding Statement declaring this commercial affiliation, as well as a statement regarding the Role of Funders in your study. If the funding organization did not play a role in the study design, data collection and analysis, decision to publish, or preparation of the manuscript and only provided financial support in the form of authors' salaries and/or research materials, please review your statements relating to the author contributions, and ensure you have specifically and accurately indicated the role(s) that these authors had in your study. You can update author roles in the Author Contributions section of the online submission form.

Please also include the following statement within your amended Funding Statement.

“The funder provided support in the form of salaries for authors [insert relevant initials], but did not have any additional role in the study design, data collection and analysis, decision to publish, or preparation of the manuscript. The specific roles of these authors are articulated in the ‘author contributions’ section.”

If your commercial affiliation did play a role in your study, please state and explain this role within your updated Funding Statement.

2. Please also provide an updated Competing Interests Statement declaring this commercial affiliation along with any other relevant declarations relating to employment, consultancy, patents, products in development, or marketed products, etc.

Within your Competing Interests Statement, please confirm that this commercial affiliation does not alter your adherence to all PLOS ONE policies on sharing data and materials by including the following statement: "This does not alter our adherence to PLOS ONE policies on sharing data and materials.” (as detailed online in our guide for authors http://journals.plos.org/plosone/s/competing-interests) . If this adherence statement is not accurate and there are restrictions on sharing of data and/or materials, please state these. Please note that we cannot proceed with consideration of your article until this information has been declared.

Please include both an updated Funding Statement and Competing Interests Statement in your cover letter. We will change the online submission form on your behalf.

5. Please upload a new copy of Figures 1, 3, and 6 as the details are not clear. Please follow the link for more information:  https://journals.plos.org/plosone/s/figures

If the reviewer comments include a recommendation to cite specific previously published works, please review and evaluate these publications to determine whether they are relevant and should be cited. There is no requirement to cite these works unless the editor has indicated otherwise.

[Note: HTML markup is below. Please do not edit.]

Reviewers' comments:

Reviewer's Responses to Questions

**Comments to the Author**

1. Is the manuscript technically sound, and do the data support the conclusions?

Reviewer #1: Partly

Reviewer #2: Partly

Reviewer #3: No

Reviewer #4: Yes

**********

2. Has the statistical analysis been performed appropriately and rigorously?

Reviewer #1: Yes

Reviewer #2: Yes

Reviewer #3: No

Reviewer #4: N/A

**********

3. Have the authors made all data underlying the findings in their manuscript fully available?

The PLOS Data policy

Reviewer #1: Yes

Reviewer #2: No

Reviewer #3: No

Reviewer #4: Yes

**********

4. Is the manuscript presented in an intelligible fashion and written in standard English?

Reviewer #1: No

Reviewer #2: Yes

Reviewer #3: No

Reviewer #4: No

**********

Reviewer #1: -The authors should revise the language

-The authors should put all the website of the tools that they used. For example, Swiss-pdb Viewer, Autodock Vina, Autodock

-line 67 in introduction, what does MDS stand for?

-the authors should explain the what are the M1, D13-L17, R69-T73, and E81-D85 region, provide citation of this explanation.

-line 83 materials and methods, Molecular Dynamic revise to Molecular Dynamics.

- Particle Mesh Ewald (PME) method explain this method and provide citation

-the authors should mention the RMSD to analyze in the materials and methods

- the authors should explain and cite the LIE method in line

- the MDS abbreviation is not very common. They used MS simulation.

-the manuscript should be organized 1- introduction, 2- materials and methods 2.2, 2.3, 2.4

3-Results and so on

-in materials and methods, the authors describe RMSF only and in Results the authors states the RMDS only. Please revise and correct these mistakes

-result line 137, how much is the binding affinities (KD) of CypA to SangfA and other inhibitors?

-results line 137, explain MDS

- figure 2 the resolution is very low. The authors should prepare a high-quality figure

-figure 3A the please explain it more

-figures 5 and 6 resolutions are very low. The authors should prepare better figures

-figure 5 caption, root mean square fluctuation abbreviation is RMSF i suggest.

- figure 7 how is prepared ? software used ?

-please discuss why is it important to inhibit CypA

-discussion line 244 ‘’ The findings are aligned with the current understanding of CypA's role and its interaction with various inhibitors and ligands’’ please provide references

-same line 245 ‘’ Prior research has established the foundation for comprehending the role of CypA and its inhibition” please provide references

- please explain all the abbreviations and mention full name in the first appearance in abstract and introduction and then abbreviation can be used consistently alone.

-language should be revised

Reviewer #2: General Assessment

The manuscript addresses an important therapeutic target, Cyclophilin A (CypA), presenting a molecular dynamics (MD) investigation of its interaction with the inhibitor Sanglifehrin A (SangfA). The topic aligns with the scope of Structural Chemistry and can be of interest to the computational and structural biology community. The use of MD simulations, RMSD/RMSF, Rg, H-bond analysis, PCA, LIE binding energy, and SASA calculations collectively offers useful insights into the structural dynamics of the CypA–SangfA interaction.

However, the manuscript requires substantial revisions before it can be considered suitable for publication. Several methodological and scientific aspects should be clarified, validated, or improved. The language and formatting need significant editing to ensure clarity and professional presentation.

________________________________________

Major Comments (Required Revisions)

1. Validation of Docking Protocol

o The manuscript uses AutoDock Vina docking but does not validate the docking (e.g., redocking the co-crystallized ligand or reporting RMSD of pose reproduction).

o Authors should include a docking validation step or justify the protocol reliability.

o While the molecular docking and molecular dynamics simulations provide valuable theoretical insights into the interaction between Cyclophilin A and Sanglifehrin A, the study remains entirely computational. To strengthen the scientific rigor and increase the impact of the work, the authors are encouraged to include—or at least propose—supporting biological or experimental validation. Validation through in vitro enzymatic inhibition assays, binding affinity measurements (e.g., SPR, ITC), or relevant antiviral/cellular activity data would help confirm the predictive findings and substantiate the therapeutic potential suggested by the computational results. Without such supporting evidence, the conclusions remain theoretical and should be interpreted with caution.

2. Force Field and Ligand Topology Accuracy

o The ligand topology is generated using PRODRG, which is known to produce suboptimal partial charges and parameters, particularly for large macrocyclic natural products like SangfA.

o The authors should justify this choice or generate ligand parameters using more accurate tools (e.g., CGenFF, GAFF/Antechamber, ATB server).

3. Insufficient Details in MD Protocol

o Starting pose selection criteria for the MD input are not clearly described.

o Simulation temperature and pressure coupling parameters are reported but missing thermostat/barostat type and coupling constants.

o Duration and conditions of the free-state ligand simulation used in LIE calculations are not clearly described.

o The current work relies solely on the LIE approach for estimating binding free energy; however, the study lacks thermodynamic free energy calculations such as MM/PBSA or MM/GBSA, which are commonly used in molecular dynamics-based binding affinity prediction. The inclusion of PBSA (Poisson–Boltzmann Surface Area) or a related method would provide a more comprehensive thermodynamic assessment of the CypA–Sanglifehrin A interaction and improve the reliability of the reported binding affinities. The authors are encouraged to perform MM/PBSA analysis or justify its omission and discuss its implications for the accuracy of the predicted binding energy.

4. Hydrogen Bond Analysis Concerns

o The manuscript states an average of 0.097±0.302 hydrogen bonds, which appears extremely low and likely indicates methodological issues or misinterpretation.

o Authors should verify hydrogen bond definitions and explore distance and angle cutoffs recommended for protein–ligand analysis.

5. Binding Free Energy Calculation and Units

o The binding free energy is reported as –85.56 ± 12.85 kJ/mol, which is extremely strong binding and requires discussion in the context of experimental values.

o There is inconsistency in energy units (kcal/mol vs kJ/mol). All values should be converted and reported consistently.

6. PCA Interpretation Requires Clarification

o PCA descriptions are unclear and contain internal inconsistencies (e.g., component contribution reporting, unclear atom numbering).

o Authors should show PC1 vs PC2 free energy landscape or porcupine plots to support claims.

7. Conclusion Claims Exceed Presented Data

o The study states implications for drug design and claims about pharmacophore relevance but does not present pharmacophore modeling or analog screening results.

o Statements about solvation preventing CypA solubility appear contradictory and should be re-evaluated scientifically.

o To better contextualize the study and support the computational findings, the authors are encouraged to cite recent related work on Cyclophilin A inhibitors and protein–ligand computational analysis, including the following relevant references: 10.3390/molecules28196859, 10.3390/cryst13091313, 10.3390/cryst13071088, and 10.1002/open.202400422. Adding these citations will strengthen the discussion and situate the study more clearly within current research in the field.

________________________________________

Minor Comments (Suggested Improvements)

1. English language requires extensive editing for grammar, conciseness, and scientific tone.

2. Multiple typos exist (e.g., “structral”, “alons”, “SagnfA”, “Md simulation”) and should be corrected.

3. Figures lack sufficient captions describing biological relevance and interaction residues.

4. References contain formatting inconsistencies and should be aligned with journal requirements.

5. Acronyms should be defined at first appearance (e.g., LIE, SASA).

6. Protein structure (PDB 1BCK) should be described including resolution, source organism, bound ligand (if any).

7. Authors should highlight how their observations compare with known CypA–SangfA

Reviewer #3: Aliabadi and Mohebbi investigated the interaction between CypA and its inhibitor. Molecular docking was used to identify the inhibitor with the highest affinity. Molecular dynamics simulations of the CypA- inhibitor complex were also performed. Binding free energy was evaluated using the linear interaction energy (LIE) method. Several metrics, including RMSD, RMSF, hydrogen bonding, and PCA, were used to characterize the conformational dynamics observed in the simulations.

Although the paper presents some interesting findings, it has fundamental issues and is not suitable for publication in its current form. The major concern is the lack of rigorous validation. There is no assessment of the robustness of the results, such as using alternative starting configurations for the MD simulations or applying additional binding free energy methods (e.g., MM-PBSA). The entire study relies on a single, relatively short (100 ns) MD simulation per system, which is insufficient and does not meet current standards.

The presentation of the work also requires substantial improvement. Conceptual and technical errors appear throughout the manuscript (e.g., Fig. 5). Overall, the study appears too preliminary for publication at this stage.

Reviewer #4: 1. How, the findings of present study important for future drug discovery targeting CypA-related diseases, authors can include a paragraph explain this aspect and novelty of their findings.

2. What are the advantages you noticed and chosen LIE as compared to other trajectory based models.

3. ΔGbind or ΔG_bind

4. “The present study performed molecular docking on several known CypA 67 inhibitors. SangfA, with the highest affinity to CypA, was used for MDS” authors can include data from their findings with other low binding energy ligands also

5. Line 68-69 needs correction

6. Lines 71-77 need to be rewritten for clarity

7. Line 78, what is the significance of performance in “Molecular Docking Performance and Analysis”

8. Line 81” the Autodock Tool was utilized for CypA ligand preparations, as described before.”, needs a reference.

9. Quality of figures is bad

10. RMSD calculations procedure need to be added to methods

11. Line 183, ΔGbind = -85.56 ± 12.85, what is the reason of fluctuations and high SD

12. In PCA analysis what authors had done to reduce dimensionality issue

13. What can be the possible reason for high SD in SASA analysis

14. doi: 10.1074/jbc.M501623200. two CypA.SFA complexes form a dimer in the crystal as well as in solution as shown by light scattering and size exclusion chromatography experiments. how this can be related to your results

**********

what does this mean?). If published, this will include your full peer review and any attached files.

If you choose “no”, your identity will remain anonymous but your review may still be made public.

**Do you want your identity to be public for this peer review?**  For information about this choice, including consent withdrawal, please see our Privacy Policy

Reviewer #1: No

Reviewer #2: No

Reviewer #3: No

Reviewer #4: No

**********

[NOTE: If reviewer comments were submitted as an attachment file, they will be attached to this email and accessible via the submission site. Please log into your account, locate the manuscript record, and check for the action link "View Attachments". If this link does not appear, there are no attachment files.]

To ensure your figures meet our technical requirements, please review our figure guidelines: https://journals.plos.org/plosone/s/figures

You may also use PLOS’s free figure tool, NAAS, to help you prepare publication quality figures: https://journals.plos.org/plosone/s/figures#loc-tools-for-figure-preparation.

NAAS will assess whether your figures meet our technical requirements by comparing each figure against our figure specifications.

# Author comment: Author response to Decision Letter 1

**DOI:** 10.1371/journal.pone.0358847.r002

5 Jun 2026

February 26, 2026

Dear Dr. Trisha Mae Tañedo Perez,

Thank you for the opportunity to revise our manuscript. We have updated the Data Availability Statement to comply with PLOS ONE policies. In our initial submission, we stated that data is available upon request; however, we have now made the core supporting information alongside the previous Table S1 file publicly available.

Specifically, we have uploaded the custom bat script developed to perform all basic GROMACS analyzes for this study. This script, along with a description of its input parameters, has been included as Supporting Information (run_basic.bat) and cited accordingly in the Methods section, ‘Trajectory Processing and Analysis Framework’. This allows readers to replicate our computational workflows fully. We have updated the manuscript to reflect this addition and have revised the Data Availability Statement to confirm that all relevant data and scripts are now publicly accessible via the manuscript's Supporting Information files.

Thank you for handling our submission.

Sincerely,

Alireza

February 13, 2026

PONE-D-25-58905

Dear Dr. Farhadul Islam, PhD

Academic Editor

PLOS ONE

Thank you for the opportunity to revise and resubmit our manuscript entitled Molecular dynamic simulation of Cyclophilin A in complex with Sanglifehrin A, which in the revised version it is changed to ‘All-Atom Molecular Dynamic Simulation of Cyclophilin A in Complex with Sanglifehrin A’. We appreciate your time, and consideration, and thoughtful comments and valuable suggestions provided by the reviewers and the editorial team. I am very sorry for the delay in providing revisions due to some internet issues we had faced. We have carefully addressed all of the concerns raised. The main changes include:

Editorial comments:

We note that one or more of the authors are employed/affiliated by a commercial company: Vista Aria Rena Gene Inc.

1. Please provide an amended Funding Statement declaring this commercial affiliation, as well as a statement regarding the Role of Funders in your study. If the funding organization did not play a role in the study design, data collection and analysis, decision to publish, or preparation of the manuscript and only provided financial support in the form of authors' salaries and/or research materials, please review your statements relating to the author contributions, and ensure you have specifically and accurately indicated the role(s) that these authors had in your study. You can update author roles in the Author Contributions section of the online submission form.

Please also include the following statement within your amended Funding Statement.

“The funder provided support in the form of salaries for authors [insert relevant initials], but did not have any additional role in the study design, data collection and analysis, decision to publish, or preparation of the manuscript. The specific roles of these authors are articulated in the ‘author contributions’ section.”

If your commercial affiliation did play a role in your study, please state and explain this role within your updated Funding Statement.

Authors’ action: The funding statement is revised as recommended to ‘The funder provided support in the form of salaries for author A.M, but did not have any additional role in the study design, data collection and analysis, decision to publish, or preparation of the manuscript. The specific roles of these authors are articulated in the ‘author contributions’ section’.

2. Please also provide an updated Competing Interests Statement declaring this commercial affiliation along with any other relevant declarations relating to employment, consultancy, patents, products in development, or marketed products, etc.

Within your Competing Interests Statement, please confirm that this commercial affiliation does not alter your adherence to all PLOS ONE policies on sharing data and materials by including the following statement: "This does not alter our adherence to PLOS ONE policies on sharing data and materials.” (as detailed online in our guide for authors http://journals.plos.org/plosone/s/competing-interests) . If this adherence statement is not accurate and there are restrictions on sharing of data and/or materials, please state these. Please note that we cannot proceed with consideration of your article until this information has been declared.

Please include both an updated Funding Statement and Competing Interests Statement in your cover letter. We will change the online submission form on your behalf.

Authors’ action: The CoI statement is revised as recommended to ‘The authors declare that the research was conducted in the absence of any commercial or financial relationships that could be construed as a potential conflict of interest. Author A.M. is employed by Vista Aria Rena Gene Inc. This does not alter our adherence to PLOS ONE policies on sharing data and materials’.

5. Please upload a new copy of Figures 1, 3, and 6 as the details are not clear. Please follow the link for more information: https://journals.plos.org/plosone/s/figures

Authors’ action: Dear editorial of PLOS ONE, we have completely renew the figures to me the journal’s criterias.

Reviewers' comments:

Review Comments to the Author

Reviewer #1: -The authors should revise the language

-The authors should put all the website of the tools that they used. For example, Swiss-pdb Viewer, Autodock Vina, Autodock

Authors’ action: Thank you for the comment we have improved the language used in the manuscript and tried to keep the terms consistent through the manuscript. Due to the depths of the comments, we have changed the title to ‘all-atom …’ to solely focus on analyzing and reporting CypA-SangfA in apo an holo systems. Therefore, all docking-relevant works have removed in the revised version.

-line 67 in introduction, what does MDS stand for?

Authors’ action: The words of ‘molecular dynamic simulation (MDS)’ was missing. Thank you for the comment. It is now placed in that line and highlighted.

-the authors should explain the what are the M1, D13-L17, R69-T73, and E81-D85 region, provide citation of this explanation.

Authors’ action: These amino acids were reported before as a binding site within CypA. We have provided a reference as recommended.

-line 83 materials and methods, Molecular Dynamic revise to Molecular Dynamics.

- Particle Mesh Ewald (PME) method explain this method and provide citation

Authors’ action: The references of PME is now added as recommended, as well as a brief clarification over using this method in MDS.

-the authors should mention the RMSD to analyze in the materials and methods

Authors’ action: Thank you for the comment. We have revised the Methods section and the RMSD analysis is explained under the sub-heading ‘Conformational Stability and Fluctuation Metrics’.

- the authors should explain and cite the LIE method in line

Authors’ action: Thank you for your comment. The reference is cited in the first line of ‘Calculation of Linear Interaction Energy (LIE)’ sub-heading at Methods section.

- the MDS abbreviation is not very common. They used MS simulation.

Authors’ action: As recommended, all MDS abbreviations changed to ‘MD simulation’.

-the manuscript should be organized 1- introduction, 2- materials and methods 2.2, 2.3, 2.4

3-Results and so on

Authors’ action: Thank you for this comment. Unfortunately, we are not able to change the manuscript style due to the journal’s guideline.

-in materials and methods, the authors describe RMSF only and in Results the authors states the RMDS only. Please revise and correct these mistakes

Authors’ action: You are correct. It is now revised and all analyzes are explained and reported in Methods and Results sections, respectively.

-result line 137, how much is the binding affinities (KD) of CypA to SangfA and other inhibitors?

Authors’ action: We have discussed binding affinity of CypA to SangfA and a well-known CypA inhibitor, Cyclosporine A (CsA). However, other inhibitors presented by the docking results are now removed.

-results line 137, explain MDS

Authors’ action: Thank you for the comment. We have now elaborated and explained more on MD simulation at the Introduction section, 4th paragraph.

- figure 2 the resolution is very low. The authors should prepare a high-quality figure

Authors’ action: Thank you. We have updated the quality of all figures for more information and new analyzes.

-figure 3A the please explain it more

Authors’ action: Thank you so much for the comment. As recommended, we have elaborated the captions of figures to reduce the size of main text.

-figures 5 and 6 resolutions are very low. The authors should prepare better figures

Authors’ action: Accordingly, we have also updated these figures in both quality and information.

-figure 5 caption, root mean square fluctuation abbreviation is RMSF i suggest.

Authors’ action: Thank you for your suggestion. According to it we have revised the caption and abbreviation are used.

- figure 7 how is prepared ? software used ?

Authors’ action: The figures are generated mainly in R and assembled in powerpoint, where we had more than one panel. The Methods section is updated accordingly.

-please discuss why is it important to inhibit CypA

Authors’ action: Thank you so much. The importance of CypA is discussed in the revised version.

-discussion line 244 ‘’ The findings are aligned with the current understanding of CypA's role and its interaction with various inhibitors and ligands’’ please provide references

Authors’ action: The sentence revised completely as a result of changes in the data.

-same line 245 ‘’ Prior research has established the foundation for comprehending the role of CypA and its inhibition” please provide references

Authors’ action: Thank you for this comment. The mentioned sentence is now cited with relevant papers, and it is now in the first line of the Discussion section in the revised version of the manuscript.

- please explain all the abbreviations and mention full name in the first appearance in abstract and introduction and then abbreviation can be used consistently alone.

Authors’ action: The appearance of the abbreviations checked in the mentioned section as correctly suggested. All the missing is now revised.

-language should be revised

Authors’ action: We’ve improved the language as recommended.

Reviewer #2: General Assessment

The manuscript addresses an important therapeutic target, Cyclophilin A (CypA), presenting a molecular dynamics (MD) investigation of its interaction with the inhibitor Sanglifehrin A (SangfA). The topic aligns with the scope of Structural Chemistry and can be of interest to the computational and structural biology community. The use of MD simulations, RMSD/RMSF, Rg, H-bond analysis, PCA, LIE binding energy, and SASA calculations collectively offers useful insights into the structural dynamics of the CypA–SangfA interaction.

However, the manuscript requires substantial revisions before it can be considered suitable for publication. Several methodological and scientific aspects should be clarified, validated, or improved. The language and formatting need significant editing to ensure clarity and professional presentation.

________________________________________

Major Comments (Required Revisions)

1. Validation of Docking Protocol

o The manuscript uses AutoDock Vina docking but does not validate the docking (e.g., redocking the co-crystallized ligand or reporting RMSD of pose reproduction).

o Authors should include a docking validation step or justify the protocol reliability.

o While the molecular docking and molecular dynamics simulations provide valuable theoretical insights into the interaction between Cyclophilin A and Sanglifehrin A, the study remains entirely computational. To strengthen the scientific rigor and increase the impact of the work, the authors are encouraged to include—or at least propose—supporting biological or experimental validation. Validation through in vitro enzymatic inhibition assays, binding affinity measurements (e.g., SPR, ITC), or relevant antiviral/cellular activity data would help confirm the predictive findings and substantiate the therapeutic potential suggested by the computational results. Without such supporting evidence, the conclusions remain theoretical and should be interpreted with caution.

Authors’ action: Thank you so much for this comment. As correctly mentioned, the molecular docking is not a weight to the work alone. Therefore, we have passed molecular docking and instead changed our work to all-atom MD simulation to deeply focus on the dynamic of CypA in apo or SangfA-bonded holo systems. We have addressed the limitation of the study and as you correctly suggested, we have included and propose experimental validation experiment and future work on drug discovery based on the novel findings of the presented study. All sections are now revised.

2. Force Field and Ligand Topology Accuracy

o The ligand topology is generated using PRODRG, which is known to produce suboptimal partial charges and parameters, particularly for large macrocyclic natural products like SangfA.

o The authors should justify this choice or generate ligand parameters using more accurate tools (e.g., CGenFF, GAFF/Antechamber, ATB server).

Authors’ action: We thank the reviewer for raising this important point. We fully acknowledge that PRODRG is not the optimal choice for generating ligand topologies for molecular dynamics simulation, and we are aware of its well-documented limitations, particularly regarding partial charge assignment and force field compatibility with modern biomolecular force fields. However, Sanglifehrin A presents exceptional challenges that made the use of standard automated parameterization tools impractical. As shown in PubChem, conformer generation for this 1090 Da macrocycle is explicitly disallowed due to its size and complexity (17 stereocenters, 15 rotatable bonds, a 19-membered bicyclic ring system). In our hands, both the ATB server and CGenFF ParamChem may fail to generate complete topologies compatible with the GROMOS 54a7 force field used in this study. Manual parameterization following CHARMM or GAFF protocols. PRODRG was therefore selected as a pragmatic compromise. We have also acknowledge the limitation of the study due to the mentioned forcefield, and we will do address this in the future study.

3. Insufficient Details in MD Protocol

o Starting pose selection criteria for the MD input are not clearly described.

o Simulation temperature and pressure coupling parameters are reported but missing thermostat/barostat type and coupling constants.

o Duration and conditions of the free-state ligand simulation used in LIE calculations are not clearly described.

o The current work relies solely on the LIE approach for estimating binding free energy; however, the study lacks thermodynamic free energy calculations such as MM/PBSA or MM/GBSA, which are commonly used in molecular dynamics-based binding affinity prediction. The inclusion of PBSA (Poisson–Boltzmann Surface Area) or a related method would provide a more comprehensive thermodynamic assessment of the CypA–Sanglifehrin A interaction and improve the reliability of the reported binding affinities. The authors are encouraged to perform MM/PBSA analysis or justify its omission and discuss its implications for the accuracy of the predicted binding energy.

Authors’ action: Thank you so much for your comment. The starting pose for the MD simulations was directly taken from the experimental crystal structure in apo, and the complex of SangfA-CypA. As recommended, information on thermostat/barostat type and coupling constants is now included in M&M. We have now revised the MS and included the duration of LIE analysis. We have updated the manuscript and included more robust and detailed analyzes and reports. Unfortunately, we were not able to conduct MM-PBSA due to the forcefield used for this study, GROMOS 54a7, that is not supported for gromacs mmpbsa. In our future work we will expand this limitation, which is already addressed in the manuscript.

4. Hydrogen Bond Analysis Concerns

o The manuscript states an average of 0.097±0.302 hydrogen bonds, which appears extremely low and likely indicates methodological issues or misinterpretation.

o Authors should verify hydrogen bond definitions and explore distance and angle cutoffs recommended for protein–ligand analysis.

Authors’ action: Thank you for this comment. The H-bond analyzes are refreshed to verify our previous miss-calculation. Of course, the number of Hydrogen bonding between SangfA and CypA are handful in the crystallized structure. This is addressed in the revised manuscript.

5. Binding Free Energy Calculation and Units

o The binding free energy is reported as –85.56 ± 12.85 kJ/mol, which is extremely strong binding and requires discussion in the context of experimental values.

o There is inconsistency in energy units (kcal/mol vs kJ/mol). All values should be converted and reported consistently.

Authors’ action: We sincerely apologize for the unit errors. The binding free energy value of –85.56 ± 12.85 was the result of LIE formula that it is now converted to –20.45 ± 3.07 kcal/mol. We have corrected this throughout the manuscript and ensured consistent use of kcal/mol for all energy reporting. The reviewer correctly notes that –20.45 kcal/mol represents an exceptionally strong binding energy that requires experimental benchmarking. The original discovery and characterization of Sanglifehrin A by Sanglier et al. (1999) established that Sanglifehrin A exhibits 10- to 20-fold higher affinity for cyclophilin A than cyclosporin A (CsA). CsA binds CypA with a Kd of approximately 6–30 nM depending on assay conditions, corresponding to a binding free energy of approximately –43 to –47 kJ/mol (–10.3 to –11.2 kcal/mol). A 10- to 20-fold improvement in Kd places the experimental ΔG for SangA at approximately –50 to –55 kJ/mol (≈ –12 to –13 kcal/mol). More recent development of SangfA analogs (https://www.biocentury.com/article/297424 has yielded compounds with Kd = 5 nM , which corresponds to ΔG ≈ –11.3 kcal/mol, consistent with the 10–20x CsA benchmark. OF course these are only for address dear reviewer concern.

6. PCA Interpretation Requires Clarification

o PCA descriptions are unclear and contain internal inconsistencies (e.g., component contribution reporting, unclear atom numbering).

o Authors should show PC1 vs PC2 free energy landscape or porcupine plots to support claims.

Authors’ action: You are correct. We did not carefully analyze and report the PCA. Therefore, we have tried to expand our analyzes on PCA and report more landscape of atomic motion and variance of the PCs.

7. Conclusion Claims Exceed Presented Data

o The study states implications for drug design and claims about pharmacophore relevance but does not present pharmacophore modeling or analog screening results.

Authors’ action: As dear reviewer recommended, the Conclusion section revised and pharmacophore modeling, virtual screening, and structure-guided drug discovery are included.

o Statements about solvation preventing CypA solubility appear contradictory and should be re-evaluated scientifically.

Authors’ action: Thank you for this comment. We have revised the sentence as recommended by including more results on the role of hydrogen bonding between ligand-protein, ligand-water, and protein-water.

o To better contextualize the study and support the computational findings, the authors are encouraged to cite recent related work on Cyclophilin A inhibitors and protein–ligand computational analysis, including the following relevant references: 10.3390/molecules28196859, 10.3390/cryst13091313, 10.3390/cryst13071088, and 10.1002/open.202400422. Adding these citations will strengthen the discussion and situate the study more clearly within current research in the field.

Authors’ action: The recommended papers are cited in appropriate places within the revised manuscript.

_____ Authors’ action: ___________________________________

Minor Comments (Suggested Improvements)

1. English language requires extensive editing for grammar, conciseness, and scientific tone.

Authors’ action: We’ve improved the revised manuscript’s grammar, conciseness, and scientific tone.

Authors’ action:

2. Multiple typos exist (e.g., “structral”, “alons”, “SagnfA”, “Md simulation”) and should be corrected.

Authors’ action: Thank you for your attention. The writing typos are addressed through the revised manuscript.

Authors’ action:

3. Figures lack sufficient captions describing biological relevance and interaction residues.

Authors’ action: As recommended, the figures’ captions revised and elaborated to reduce the main text size and improve the quality reporting data.

Authors’ action:

4. References contain formatting inconsistencies and should be aligned with journal requirements.

Authors’ action: The references are aligned with the PLOS ONE’s guideline now.

Authors’ action:

5. Acronyms should be defined at first appearance (e.g., LIE, SASA).

Authors’ action: The acronyms are addressed as recommended.

6. Protein structure (PDB 1BCK) should be described including resolution, source organism, bound ligand (if any).

Authors’ action: As recommended, more information is included in the ‘Preparation of CypA Crystal Structure and its complex with SangfA’ section.

Authors’ action:

7. Authors should highlight how their observations compare with known CypA–SangfA

Authors’ action: We have discussed Cyclosporin A, but due to the current size of the revised manuscript we were not able to discuss more, but rather we discussed our results.

Reviewer #3: Aliabadi and Mohebbi investigated the interaction between CypA and its inhibitor. Molecular docking was used to identify the inhibitor with the highest affinity. Molecular dynamics simulations of the CypA- inhibitor complex were also performed. Binding free energy was evaluated using the linear interaction energy (LIE) method. Several metrics, including RMSD, RMSF, hydrogen bonding, and PCA, were used to characterize the conformational dynamics observed in the simulations.

Although the paper presents some interesting findings, it has fundamental issues and is not suitable for publication in its current form. The major concern is the lack of rigorous validation. There is no assessment of the robustness of the results, such as using alternative starting configurations for the MD simulations or applying additional binding free energy methods (e.g., MM-PBSA). The entire study relies on a single, relatively short (100 ns) MD simulation per system, which is insufficient and does not meet current standards.

Authors’ action: Thank you so much for your comment. We have updated the manuscript and included more robust and detailed analyzes and reports. Unfortunately, we were not able to conduct MM-PBSA due to the forcefield used for this study, GROMOS 54a7, that is not supported for gromacs mmpbsa. In our future work we will expand this limitation, which is already addressed in the manuscript.

The presentation of the work also requires substantial improvement. Conceptual and technical errors appear throughout the manuscript (e.g., Fig. 5). Overall, the study appears too preliminary for publication at this stage.

Authors’ action: As you’ve correctly mentioned, figures needed lots of works and attentions. We have performed a more comprehensive works and detailed statistical analyzes to report higher-quality of data both in the form of tables and figures.

Reviewer #4: 1. How, the findings of present study important for future drug discovery targeting CypA-related diseases, authors can include a paragraph explain this aspect and novelty of their findings.

Authors’ action: Thank you for this important comment. Cyclophilin A is implicated in viral replication like HIV-1, HCV, Influenza A virus, inflammatory signaling, cancer progression, and immune regulation. Therefore, selective modulation of CypA–ligand interactions represent an attractive therapeutic strategy. Our simulations provide atomistic insight into the stability, key hydrogen bonds, hydrophobic hotspots, and energetic contributions governing binding of Sanglifehrin A derivatives. Identification of dominant residues and favorable interaction patterns enables rational scaffold optimization and structure-guided design of next-generation CypA inhibitors with improved affinity and specificity. We have revised the Discussion for more detail on this matter.

2. What are the advantages you noticed and chosen LIE as compared to other trajectory based models.

Authors’ action: The LIE method was selected because it provides a favorable balance between computational efficiency and accuracy while allowing extensive sampling across multiple replicas. Compared with more computationally demanding approaches such as FEP/TI, LIE enables rapid evaluation of multiple systems and long trajectories, which is advantageous for screening studies. In contrast to endpoint methods alone, LIE explicitly incorporates averaged electrostatic and van der Waals interaction energies along the trajectory, capturing dynamic effects. As we have mentioned earlier, we would have conduct a MM-PBSA calculations that was unfortunately not supported our used forcefield.

3. ΔGbind or ΔG_bind

Authors’ action: We have revised the manuscript and made a consistent use of the word ΔGbind.

4. “The present study performed molecular docking on several known CypA 67 inhibitors. SangfA, with the highest affinity to CypA, was used for MDS” authors can include data from their findings with other low binding energy ligands also

5. Line 68-69 needs correction

6. Lines 71-77 need to be rewritten for clarity

7. Line 78, what is the significance of performance in “Molecular Docking Performance and Analysis”

Authors’ action: Thank you so much for this comment. Since th original manuscript was mainly covered MD simulation of CypA complexed SangfA, we’ve changed the scope of the study to remove docking results and pay more attention to all-atom MD simulation of CypA upon SangfA bonding. All mentioned sentences are not revised.

8. Line 81” the Autodock Tool was utilized for CypA ligand preparations, as described before.”, needs a reference.

Authors’ action: The references are included in the last sentence at ‘Preparation of CypA Crystal Structure and its complex with SFA’ subheading.

9. Quality of figures is bad

Authors’ action: The figures are updated as correctly recommended.

10. RMSD calculations procedure need to be added to methods

Authors’ action: The method section revised as dear reviewer correctly recommended, and analyzes are added in more details.

11. Line 183, ΔGbind = -85.56 ± 12.85, what is the reason of fluctuations and high SD

Authors’ action: Since we had reported the LIE energy as Kj/mol, in the revised version we’ve converted the value to Kcal/mol for the sake of consistency and the result is now -20.45 ± 3.07 Kcal/mol.

12. In PCA analysis what authors had done to reduce dimensionality issue

Authors’ action: Thank you so much. We have sampled first 20 PCs to evaluate the variance of atomic fluctuations during the time of simulation. In the first draft of the manuscript, this may not well conveyed. Therefore, we have now updated the PCA reports both in more details in M&M section and Results section.

13. What can be the possible reason for high SD in SASA analysis

Authors’ action: In this updated revised version of our study, the observed increase in SASA may reflect the protein's overall solvent exposure, potentially through slight rearrangements of peripheral loops or side chains. We have also, included this in the Results section that was in accord with the structural findings.

14. doi: 10.1074/jbc.M501623200. two CypA.SFA complexes form a dimer in the crystal as well as in solution as shown by light scattering and size exclusion chromatography experiments. how this can be related to your results

Authors’ action: Thank you for this important notion. Consistent with the crystallographic CypA–SangfA complex in the mentioned study, the ligand remains deeply embedded; however, MD reveals that the six static hydrogen bonds resolve into a dynamic ensemble of transient contacts. Our simulations both corroborate and extend previous structural and biochemical studies of the Cyclophilin A–Sanglifehrin A complex. While the crystallographic model reported by Kallen et al. depicts a tightly hydrogen-bonded ligand deeply embedded in the pocket, our trajectories reveal that these interactions are highly dynamic, with transient, solvent-mediated contacts maintaining stable binding. Consistent with Eisenmesser et al.’s identification of the L98–S99 region as a catalytically mobile loop, SangfA induces β-strand formation and local rigidification in this segment, providing a mechanistic explanation for enzymatic inhibition. Principal component and clustering analyses further support a conformational selection model, as the holo ensemble explores a broader yet redistributed landscape with increased configurational entropy. Together, these results show that SangfA inhibits CypA not by rigid locking but by selectively reshaping its dynamic ensemble. This is now discussed in Discussion section at 2nd paragraph.

Again, we are appreciating the time, consideration, and the editorials and dear reviewers’ comments that helped us to improve the manuscript in different aspects. I hope the revised manuscript met the journals and reviewers’ standards.

Bests

Alireza Mohebbi

Ph.D. student at Iran University of Medical Sciences, Tehran, Iran

Email: Alirezaa2s@gmail.com Tel: +98 935 467 4593

Attachment Submitted filename: response_to_reviewers_.docx

# Peer review: Decision Letter 1

**DOI:** 10.1371/journal.pone.0358847.r003

**Authors:** Farhadul Islam

27 Jul 2026

Dear Dr. Mohebbi,

Thank you for submitting your manuscript to PLOS ONE. After careful consideration, we feel that it has merit but does not fully meet PLOS ONE’s publication criteria as it currently stands. Therefore, we invite you to submit a revised version of the manuscript that addresses the points raised during the review process.

Please submit your revised manuscript by Sep 10 2026 11:59PM. If you will need more time than this to complete your revisions, please reply to this message or contact the journal office at plosone@plos.org. When you're ready to submit your revision, log on to https://www.editorialmanager.com/pone/ and select the 'Submissions Needing Revision' folder to locate your manuscript file.

- A letter that responds to each point raised by the academic editor and reviewer(s). You should upload this letter as a separate file labeled 'Response to Reviewers'.
- A marked-up copy of your manuscript that highlights changes made to the original version. You should upload this as a separate file labeled 'Revised Manuscript with Track Changes'.
- An unmarked version of your revised paper without tracked changes. You should upload this as a separate file labeled 'Manuscript'.

If you would like to make changes to your financial disclosure, please include your updated statement in your cover letter. Guidelines for resubmitting your figure files are available below the reviewer comments at the end of this letter.

If applicable, we recommend that you deposit your laboratory protocols in protocols.io to enhance the reproducibility of your results. Protocols.io assigns your protocol its own identifier (DOI) so that it can be cited independently in the future. For instructions see: https://journals.plos.org/plosone/s/submission-guidelines#loc-laboratory-protocols. Additionally, PLOS ONE offers an option for publishing peer-reviewed Lab Protocol articles, which describe protocols hosted on protocols.io. Read more information on sharing protocols at https://plos.org/protocols?utm_medium=editorial-email&utm_source=authorletters&utm_campaign=protocols.

As the corresponding author, your ORCID iD is verified in the submission system and will appear in the published article. PLOS supports the use of ORCID, and we encourage all coauthors to register for an ORCID iD and use it as well. Please encourage your coauthors to verify their ORCID iD within the submission system before final acceptance, as unverified ORCID iDs will not appear in the published article. *Only*  the individual author can complete the verification step; PLOS staff *cannot*  verify ORCID iDs on behalf of authors.

We look forward to receiving your revised manuscript.

Kind regards,

Farhadul Islam, PhD

Academic Editor

PLOS One

Journal Requirements:

If the reviewer comments include a recommendation to cite specific previously published works, please review and evaluate these publications to determine whether they are relevant and should be cited. There is no requirement to cite these works unless the editor has indicated otherwise.

Please review your reference list to ensure that it is complete and correct. If you have cited papers that have been retracted, please include the rationale for doing so in the manuscript text, or remove these references and replace them with relevant current references. Any changes to the reference list should be mentioned in the rebuttal letter that accompanies your revised manuscript. If you need to cite a retracted article, indicate the article’s retracted status in the References list and also include a citation and full reference for the retraction notice.

Additional Editor Comments:

Thanks for submitting the revised manuscript and the revised manuscript improved notably. However, it needs to address some minor comments of the reviewers, especially reviewer #5.

[Note: HTML markup is below. Please do not edit.]

Reviewers' comments:

Reviewer's Responses to Questions

**Comments to the Author**

Reviewer #1: All comments have been addressed

Reviewer #2: All comments have been addressed

Reviewer #4: All comments have been addressed

Reviewer #5: (No Response)

**********

2. Is the manuscript technically sound, and do the data support the conclusions?

Reviewer #1: Yes

Reviewer #2: Yes

Reviewer #4: Yes

Reviewer #5: Yes

**********

3. Has the statistical analysis been performed appropriately and rigorously?

Reviewer #1: I Don't Know

Reviewer #2: Yes

Reviewer #4: N/A

Reviewer #5: Yes

**********

4. Have the authors made all data underlying the findings in their manuscript fully available?

The PLOS Data policy

Reviewer #1: Yes

Reviewer #2: Yes

Reviewer #4: Yes

Reviewer #5: No

**********

5. Is the manuscript presented in an intelligible fashion and written in standard English?

Reviewer #1: Yes

Reviewer #2: Yes

Reviewer #4: Yes

Reviewer #5: Yes

**********

Reviewer #1: Dear authors

Thank you very much for addressing all my questions.

-the authors should recommend NMR or x ray crystallographic studies to verify their findings

-the tables and figures should be cited in the discussion in my opinion.

Reviewer #2: Yes. The manuscript is generally intelligible and written in understandable English. However, minor typographical and grammatical errors remain and should be corrected during final editorial polishing.

Reviewer #4: as on revision, manuscript is quite polished and can be considered for publication upon minor checks and reading

Reviewer #5: All-Atom Molecular Dynamic Simulation of Cyclophilin A in Complex with Sanglifehrin A

The authors investigate the interactions between Cyclophilin A and Sanglifehrin A using Molecular Dynamics simulation. The manuscript has a number of minor issues that make it difficult to follow the findings reported. The analyses are interesting and they could contribute to refreshing the methodology of the field. I would ask the authors to address several concerns before recommending the publication of this work.

Please provide a figure with the most representative structures found during the simulations, pointing out relevant domains and residues.

Please provide an animation or some other means to visualize the motions associated with the main principal components. Examples can be found in

Martínez-Archundia, M., Correa-Basurto, J., Montaño, S., & Rosas-Trigueros, J. L. (2019). Studying the collective motions of the adenosine A2A receptor as a result of ligand binding using principal component analysis. Journal of Biomolecular Structure and Dynamics. https://doi.org/10.1080/07391102.2018.1564700

In line 95: The system was then neutralized by Clor Na+ ions.

Please report the nature and number of the ions used.

In line 128, Essential Dynamics and Conformational Sampling

Equations should be numbered and discussed in the text.

They write “The first few principal components (PCs) with the largest eigenvalues (> 80%)”. Please state how many PCs were used and better explain the 80% criterion.

In line 150 Calculation of Linear Interaction Energy (LIE),

Same problem with the equations. Their reference [51] does a good job writing these equations, they should follow this reference more closely.

I’m confused. In line 510 they write “complementing Asp55’s anchoring of the ligand. Yet because Arg55…”. So, is residue 55 Asp or Arg?

Other minor issues follow:

15 Beside basid MD analyzes… basic MD analyses ?

45 escalated the opportunistic… escalated an opportunistic

In line 13 they define molecular dynamics (MD), and later in line 66 they re-define molecular dynamic (MD)... It is my perception that the most accepted variation is Molecular Dynamics Simulations, either as MD or MDS.

97 at 300°K… at 300 K

111 implemented to compared… implemented to compare

484 Teses indicate that ligand binding… Consider rewriting.

**********

what does this mean?). If published, this will include your full peer review and any attached files.

If you choose “no”, your identity will remain anonymous but your review may still be made public.

**Do you want your identity to be public for this peer review?**  For information about this choice, including consent withdrawal, please see our Privacy Policy

Reviewer #1: No

Reviewer #2: **Yes:** Ahmed Hassan Bakheit

Reviewer #4: No

Reviewer #5: No

**********

[NOTE: If reviewer comments were submitted as an attachment file, they will be attached to this email and accessible via the submission site. Please log into your account, locate the manuscript record, and check for the action link "View Attachments". If this link does not appear, there are no attachment files.]

To ensure your figures meet our technical requirements, please review our figure guidelines: https://journals.plos.org/plosone/s/figures

You may also use PLOS’s free figure tool, NAAS, to help you prepare publication quality figures: https://journals.plos.org/plosone/s/figures#loc-tools-for-figure-preparation.

NAAS will assess whether your figures meet our technical requirements by comparing each figure against our figure specifications.

# Author comment: Author response to Decision Letter 2

**DOI:** 10.1371/journal.pone.0358847.r004

4 Sep 2026

September 4, 2026

Manuscript Title: All-Atom Molecular Dynamic Simulation of Cyclophilin A in Complex with Sanglifehrin A

Dear Editorial of PLOS ONE,

Thank you and the reviewers for the careful review of our manuscript and for the opportunity to revise our manuscript. Below, we have addressed the reviewers concerns and recommendations thoroughly point-by-point.

Reviewer 1

Comment: "Dear authors, Thank you very much for addressing all my questions. The authors should recommend NMR or X-ray crystallographic studies to verify their findings. The tables and figures should be cited in the discussion in my opinion."

Authors' Response: We thank Reviewer for their positive assessment and constructive suggestions. We have incorporated both recommendations into the revised manuscript:

1. Recommendation of experimental validation: We have added a paragraph in the Discussion section explicitly recommending NMR and X-ray crystallographic studies to experimentally validate our computational predictions. We now state:

" Future NMR and X-ray crystallographic experiments can be conducted to validate the predicted conformational switch in the β2-β3 loop on the CypA-SangfA complex would be invaluable. Especially, the findings on the transition of residues Ile97-Asn10 from coil in apo system to a β-sheet in holo system warrants further experimental validation. Additionally, structure-guided design, pharmacophore modeling, virtual screening, and synthesis of SangfA analogs that contact with Lys105 or that reinforce the coil-to-sheet transition in the 100-111 segment could yield inhibitors with tailored dynamic profiles and improved pharmacological properties."

2. Citation of tables and figures in the Discussion: We have carefully revised the Discussion section to ensure that tables or figures are cited where it was possible.

Reviewer 2

Comment: "Yes. The manuscript is generally intelligible and written in understandable English. However, minor typographical and grammatical errors remain and should be corrected during final editorial polishing."

Authors' Response: We thank dear Reviewer for careful reading. We have performed a thorough proofreading of the entire manuscript and corrected all identified typographical and grammatical errors. Specific corrections include:

• Line 15: "basid MD analyzes" → "basic MD analyses"

• Line 45: "escalated an opportunistic viral and bacterial co-infection" → "escalated an opportunistic viral and bacterial co-infection" (corrected)

• Line 66: Removed redundant re-definition of "molecular dynamics (MD)"

• Line 97: "at 300°K" → "at 300 K"

• Line 111: "implemented to compared" → "implemented to compare"

• Line 484: "Teses indicate that ligand binding..." → "These results indicate that ligand binding..."

We have also conducted a comprehensive language polish throughout the manuscript to improve clarity and readability.

Reviewer 4

Comment: "As on revision, manuscript is quite polished and can be considered for publication upon minor checks and reading."

Authors' Response: We thank Reviewer 4 for their positive assessment and recommendation for publication. We have performed a final careful reading and formatting check of the entire manuscript to ensure all minor issues are resolved.

Reviewer 5

Comment 1: "Please provide a figure with the most representative structures found during the simulations, pointing out relevant domains and residues."

Authors' Response: We thank Reviewer for this valuable suggestion. We have added two new figure (Figure 8 and Figure 9) to the revised manuscript that presents the most representative structures from the simulations, with relevant domains and residues clearly labeled.

We have also updated the figure legend to provide full details on the structural features shown and their significance.

Comment 2: "Please provide an animation or some other means to visualize the motions associated with the main principal components. Examples can be found in Martínez-Archundia, M., Correa-Basurto, J., Montaño, S., & Rosas-Trigueros, J. L. (2019). Studying the collective motions of the adenosine A2A receptor as a result of ligand binding using principal component analysis. Journal of Biomolecular Structure and Dynamics."

Authors' Response: We sincerely thank Reviewer #5 for this valuable suggestion. We agree that visualizing the protein motions provides crucial insight into the conformational dynamics described in our study. In response to this comment, we have generated a supplementary video (Supplementary Videos S1) from our MD trajectories that directly address this request:

Direct visualization of PC1 and PC2 motions: The dominant collective motions identified through PCA correspond to the large-amplitude conformational rearrangements that are clearly visible in these animations.

However, the β2-β3 loop, which undergoes a coil-to-β-strand transition upon ligand binding is now presented in Figure 8 and Figure 9, allowing the reader to correlate the secondary structure changes with the observed dynamics.

Comment 3: "In line 95: The system was then neutralized by Cl⁻ or Na⁺ ions. Please report the nature and number of the ions used."

Authors' Response: We apologize for this omission. The revised manuscript now explicitly states the nature and number of ions used for neutralization. In the Methods section, we now write:

"Both apo and holo systems were then neutralized by adding 2 Cl- ions …"

Comment 4: "In line 128, Essential Dynamics and Conformational Sampling. Equations should be numbered and discussed in the text."

Authors' Response: We have revised the Essential Dynamics section to ensure all equations are properly numbered and discussed in the text. Further explanation of each equation is not included.

Comment 5: "They write 'The first few principal components (PCs) with the largest eigenvalues (>80%)'. Please state how many PCs were used and better explain the 80% criterion."

Authors' Response: We thank the reviewer for catching this ambiguity. In the revised manuscript, we now write:

"The first four principal components (PCs) with the largest eigenvalues were computed, capturing 83.42% …”.

Comment 6: "In line 150 Calculation of Linear Interaction Energy (LIE). Same problem with the equations. Their reference [51] does a good job writing these equations, they should follow this reference more closely."

Authors' Response: We have thoroughly revised the LIE section to follow the formalism of reference [51] (Rifai et al., 2020) more closely. The revised Methods section now presents the equations in a clearer form:

We have added explanatory text following each equation to clarify the physical meaning of each term.

Comment 7: "I'm confused. In line 510 they write 'complementing Asp55's anchoring of the ligand. Yet because Arg55...'. So, is residue 55 Asp or Arg?"

Authors' Response: We sincerely apologize for this typographical error and the confusion it caused. Residue 55 in Cyclophilin A is Arginine (Arg55), as correctly identified in our contact analysis reports and structural data (Table S1, Table S3, and residue-level contact analysis). The mention of "Asp55" was an unintentional typo; it should have read "Arg55" throughout that sentence. We have corrected the manuscript accordingly.

We appreciate the reviewer's diligence in catching this error.

Comment 8: "Other minor issues follow: 15 Beside basid MD analyzes... basic MD analyses?"

Authors' Response: We apologize for these typographical errors. In the revised manuscript

Comment 9: "45 escalated the opportunistic... escalated an opportunistic"

Authors' Response: Corrected. The revised manuscript (page 3, line 45) now reads:

"It has been reported that CypA escalated an opportunistic viral and bacterial co-infection [16]."

Comment 10: "In line 13 they define molecular dynamics (MD), and later in line 66 they re-define molecular dynamic (MD)... It is my perception that the most accepted variation is Molecular Dynamics Simulations, either as MD or MDS."

Authors' Response: We thank the reviewer for this clarification. We have revised the manuscript. The redundant re-definition was removed at line 66, which now simply uses "MD simulation" without re-defining the abbreviation.

We have also used the full term "Molecular Dynamics Simulations" consistently throughout the manuscript where appropriate.

Comment 11: "97 at 300°K... at 300 K"

Authors' Response: Corrected. The revised manuscript now states:

Comment 12: "111 implemented to compared... implemented to compare"

Authors' Response: Corrected. The revised manuscript now reads:

"...implemented to compare the holo form …"

Comment 13: "484 Teses indicate that ligand binding... Consider rewriting."

Authors' Response: We have rewritten this sentence for clarity.

Additional Editor Comments

Editor Comment: "Thanks for submitting the revised manuscript and the revised manuscript improved notably. However, it needs to address some minor comments of the reviewers, especially reviewer #5."

Authors' Response: We thank the Academic Editor for the positive assessment and for the opportunity to further improve our manuscript. We have addressed all comments raised by dear Reviewers in detail, as described above.

Journal Requirements

"Please review your reference list to ensure that it is complete and correct. If you have cited papers that have been retracted, please include the rationale for doing so in the manuscript text, or remove these references and replace them with relevant current references."

Authors' Response: We have carefully reviewed our complete reference list. All references are current, correct, and properly formatted. We have confirmed that none of the cited papers have been retracted.

We hope that these revisions adequately address all comments raised by the reviewers and the Academic Editor. We believe the manuscript is now significantly improved and suitable for publication in PLOS ONE. We thank all reviewers for their constructive feedback, which has helped us strengthen the clarity, rigor, and completeness of our work.

Corresponding Author: Alireza Mohebbi, PhD

Email: alirezaa2s@gmail.com

Attachment Submitted filename: response_to_reviewers.docx

# Editor report: Decision Letter 2

**DOI:** 10.1371/journal.pone.0358847.r005

**Authors:** Farhadul Islam

7 Sep 2026

All-Atom Molecular Dynamics Simulation of Cyclophilin A in Complex with Sanglifehrin A

PONE-D-25-58905R2

Dear Dr. Mohebbi,

We’re pleased to inform you that your manuscript has been judged scientifically suitable for publication and will be formally accepted for publication once it meets all outstanding technical requirements.

Within one week, you’ll receive an e-mail detailing the required amendments. When these have been addressed, you’ll receive a formal acceptance letter and your manuscript will be scheduled for publication.

An invoice will be generated when your article is formally accepted. Please note, if your institution has a publishing partnership with PLOS and your article meets the relevant criteria, all or part of your publication costs will be covered. Please make sure your user information is up-to-date by logging into Editorial Manager at Editorial Manager® and clicking the ‘Update My Information' link at the top of the page. For questions related to billing, please contact billing support.

If your institution or institutions have a press office, please notify them about your upcoming paper to help maximize its impact. If they’ll be preparing press materials, please inform our press team as soon as possible -- no later than 48 hours after receiving the formal acceptance. Your manuscript will remain under strict press embargo until 2 pm Eastern Time on the date of publication. For more information, please contact onepress@plos.org.

Kind regards,

Farhadul Islam, PhD

Academic Editor

PLOS One

Additional Editor Comments (optional):

Reviewers' comments:

# Editor report: Acceptance letter

**DOI:** 10.1371/journal.pone.0358847.r006

**Authors:** Farhadul Islam

PONE-D-25-58905R2

PLOS One

Dear Dr. Mohebbi,

I'm pleased to inform you that your manuscript has been deemed suitable for publication in PLOS One. Congratulations! Your manuscript is now being handed over to our production team.

At this stage, our production department will prepare your paper for publication. This includes ensuring the following:

* All references, tables, and figures are properly cited

* All relevant supporting information is included in the manuscript submission,

* There are no issues that prevent the paper from being properly typeset

You will receive further instructions from the production team, including instructions on how to review your proof when it is ready. Please keep in mind that we are working through a large volume of accepted articles, so please give us a few days to review your paper and let you know the next and final steps.

Lastly, if your institution or institutions have a press office, please let them know about your upcoming paper now to help maximize its impact. If they'll be preparing press materials, please inform our press team within the next 48 hours. Your manuscript will remain under strict press embargo until 2 pm Eastern Time on the date of publication. For more information, please contact onepress@plos.org.

You will receive an invoice from PLOS for your publication fee after your manuscript has reached the completed accept phase. If you receive an email requesting payment before acceptance or for any other service, this may be a phishing scheme. Learn how to identify phishing emails and protect your accounts at https://explore.plos.org/phishing.

If we can help with anything else, please email us at customercare@plos.org.

Thank you for submitting your work to PLOS One and supporting open access.

Kind regards,

PLOS One Editorial Office Staff

on behalf of

Dr Farhadul Islam

Academic Editor

PLOS One
